# Supplementary material for: Measuring Child Labor: The Who’s, the Where’s, the When’s, and the Why’s
Source: PLoS One. 2025 Jun 9;20(6):e0322987. doi: 10.1371/journal.pone.0322987 (PMC12148184; doi:10.1371/journal.pone.0322987)
Supplement: SI Text — (PDF) [file pone.0322987.s001.pdf]

Additional details on classification methodologies

This Appendix compiles additional details on how the International Labor Organization (ILO), NORC and ENVERITAS compile data on children' work hours and work conditions, and on how these data is used to compute children in employment and child labor statistics in each case.

ILO

Figure S1: Conceptual framework of the ILO global estimation of child labor

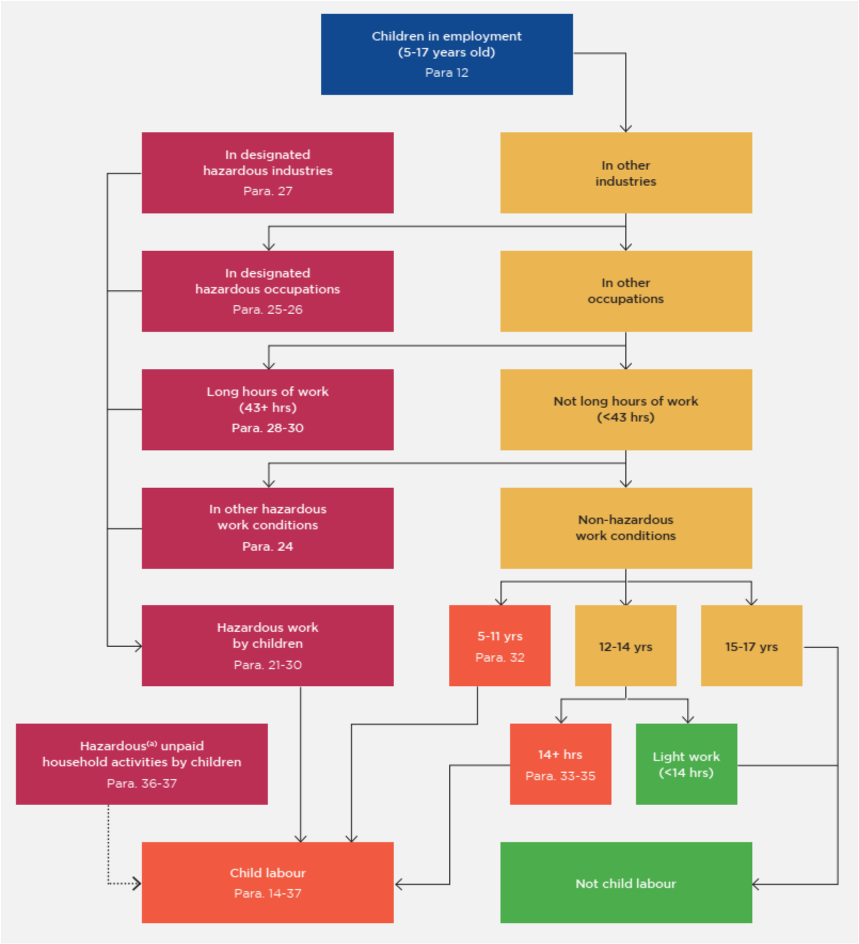

Notes: extracted from [1].

NORC

Figure S2: Conceptual framework of the NORC estimation of child labor in cocoa farming

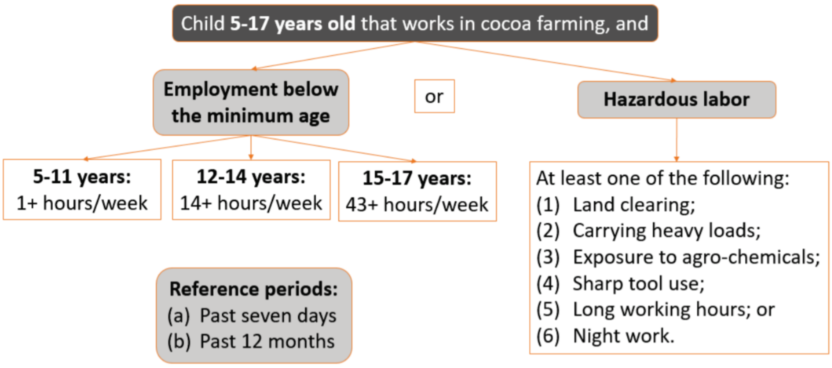

Notes:

ENVERITAS

Figure S3: Geographical coverage of ENVERITAS data

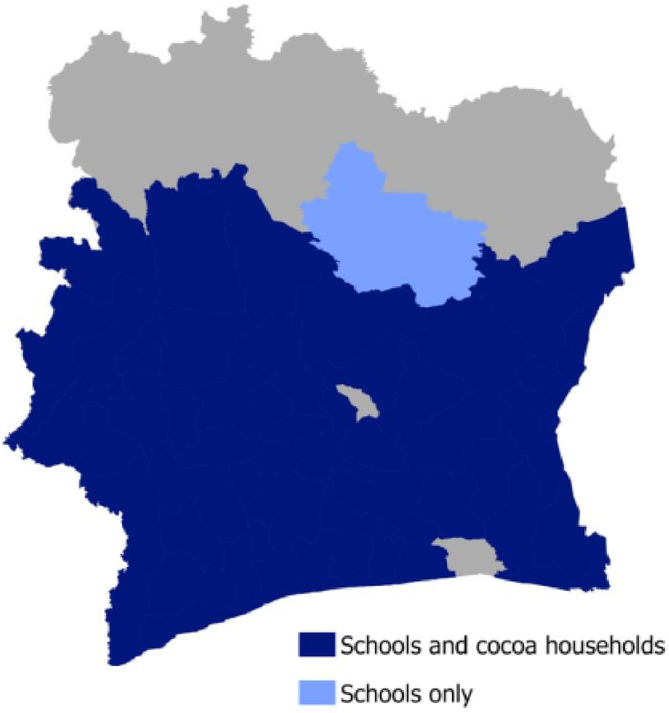

Notes: Elaborated by the authors.

Figure S4: Satellite picture of a community visited by ENVERITAS

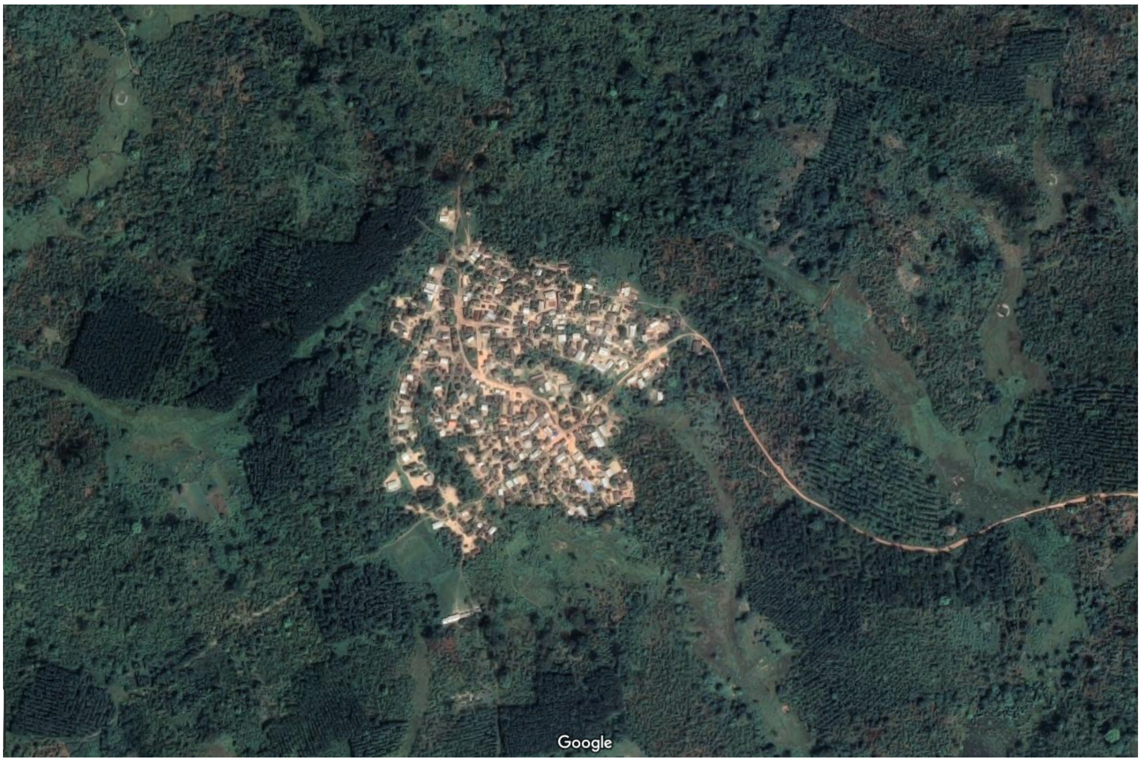

Notes: Satellite picture of Baho-Brousse, a community visited by ENVERITAS on Jan/2020. The surrounding cocoa fields are clearly visible in the picture (those not arranged in rows, which, in turn, are rubber plantations).

Figure S5: Enumerators surveying hard-to-reach communities (Jan/2020)

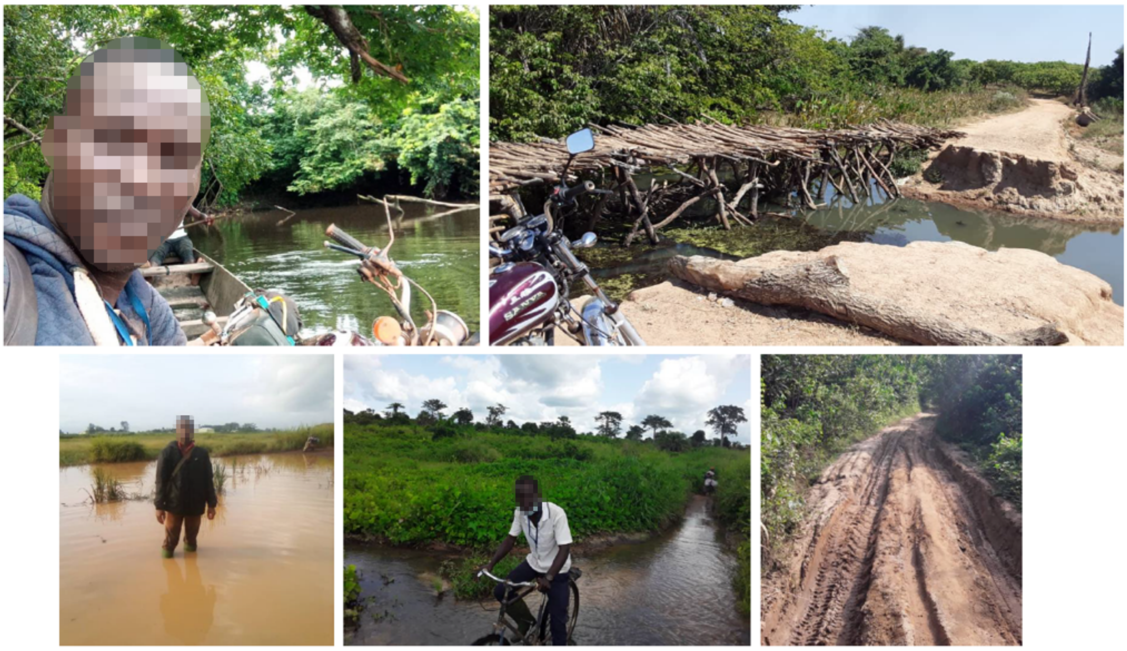

Figure S6: Enumerators surveying hard-to-reach communities (Jan/2020)

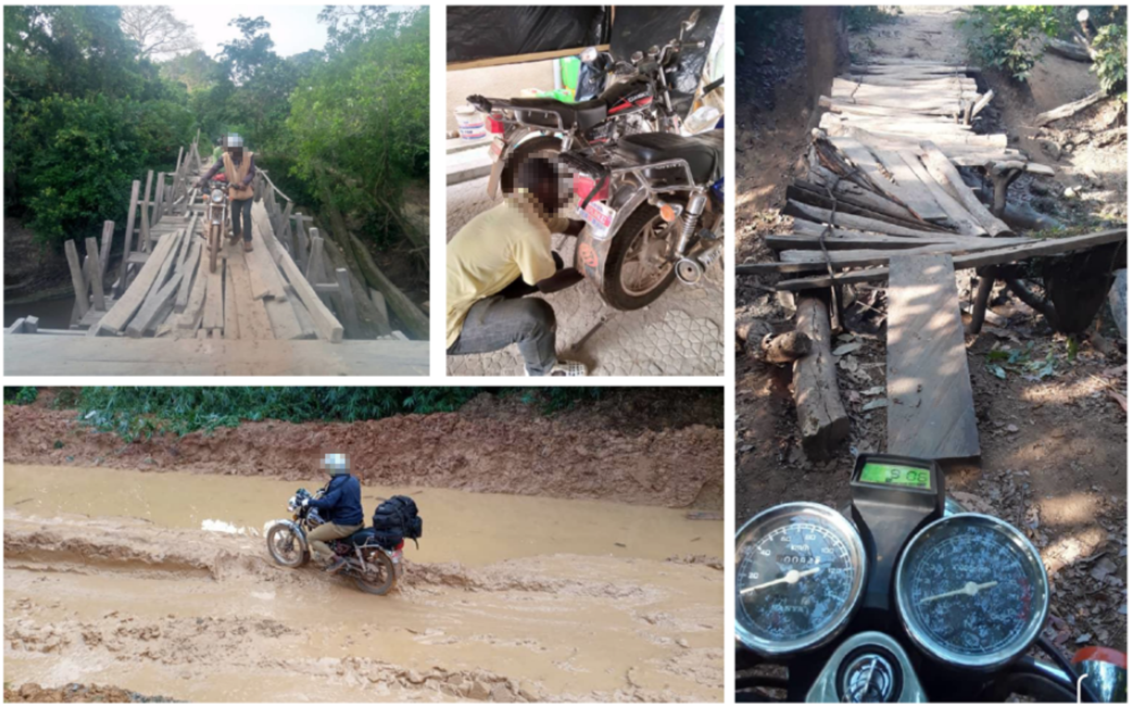

Survey instruments

This Appendix compiles the questions used to assess children in employment through our business-as-usual surveys, organized by wave and by whom was asked in each case. For all details on the survey instruments used in the context of the over-arching project, see [15].

Table S1: Survey questions about children in employment

Questions	Timeline	Respondent
"In the last month, have you engaged in one or more of the following activities, for one hour or more? Work in a cocoa plantation"	Baseline	Children
"I will now ask you some questions about activities that your children might have recently performed. In the last month, has any of your children engaged in one or more of the following activities, for one hour or more? Work in a cocoa plantation"	Baseline	Parents
"In the last month, have you engaged in one or more of the following activities, for one hour or more? Work in a cocoa plantation"	End line	Children
"I will now ask you some questions about activities that your children might have recently performed. In the last month, has any of your children engaged in one or more of the following activities, for one hour or more? Work in a cocoa plantation"	End line	Parents
"Did (child name) work in cocoa fields more than 10 hours a week in a typical week last year?"	Follow-up	Parents
"Did (child name) work in cocoa fields more than 10 hours a week during holidays last year?"	Follow-up	Parents
"To the best of your knowledge in this list of students, can you point out the students who worked in cocoa fields over 10 hours a week in a typical week last year?"	Follow-up	Teachers
"To the best of your knowledge in this list of students, can you point out the students who worked in cocoa fields over 10 hours a week in a typical week during vacation last year?"	Follow-up	Teachers
"To the best of your knowledge in this list of students, can you point out the students who worked at all in cocoa fields during the school year last year?"	Follow-up	Teachers

Notes:

Descriptive statistics

This Appendix compiles descriptive statistics of the study sample, based on our baseline survey. Table S2 provides summary statistics for student and household characteristics. Figure S7 showcases the share of children in employment by student and household characteristics. Next, Figure S8 displays classroom-level correlations between the baseline share of children in employment (based on children's self-reports) and baseline standardized test scores (Panel A) and student dropout rates by the end of the school year (Panel B; restricting attention the control group of the intervention). Figure S9 displays the histogram of households' distance to the school where their children were enrolled at the time of the survey (the measure of remoteness we explore in the main text). Last, Figure S10 showcases the prevalence of children in employment by activity and reporting source.

Table S2: Descriptive statistics (baseline survey)

	Mean	S.D.	Obs
Child is a girl	0.50	0.50	2,475
Child age			2,150
Under 5 years old	0.00	0.04	
5-11 years old	0.92	0.27	
12-14 years old	0.07	0.26	
15 years old and above	0.01	0.08	
Enrolled in 1st primary cycle (CP2)	0.52	0.50	2,475
Rural household	0.50	0.50	2,471
Household monthly income (in 2015 USD)			2,177
Less than USD 19	0.06	0.24	
USD 19-37	0.16	0.37	
USD 37-55	0.16	0.36	
USD 55-92	0.21	0.41	
USD 92-185	0.23	0.42	
USD 185-370	0.11	0.31	
More than USD 370	0.07	0.26	

Notes: CP2 is the second grade for the 1st primary cycle in Côte D'Ivoire education. Rural areas are defined according to parents' main occupation: agricultural or plantation activities are defined as rural. Household monthly income was reported in CFCA and converted to 2015 USD.

Figure S7: Share of students who worked for at least one hour in cocoa plantations over the last month, according to children

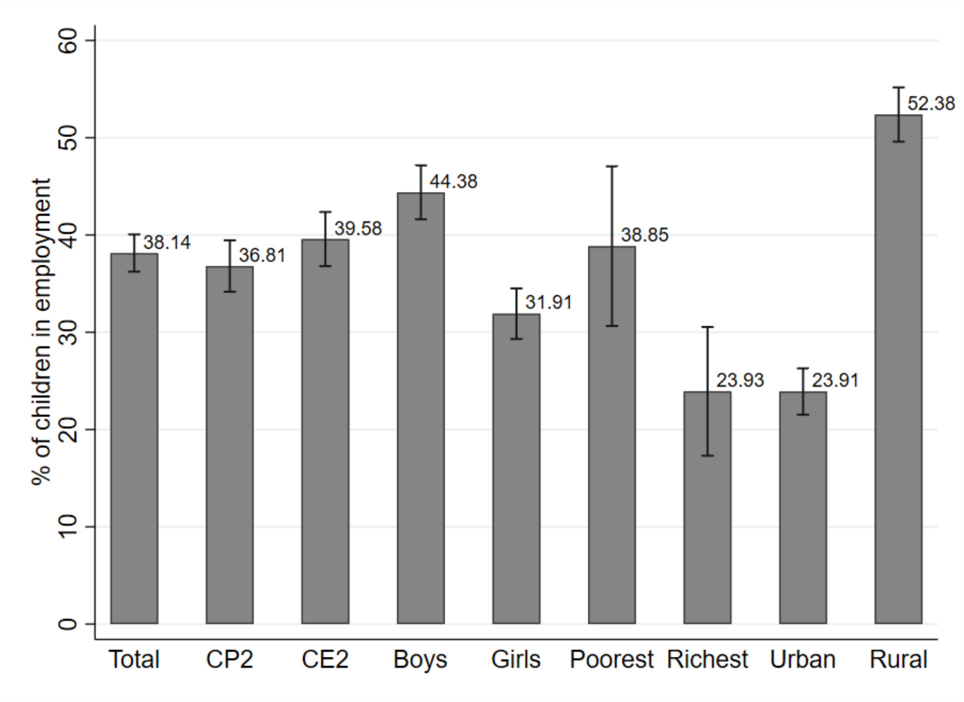

Notes: Figure S7 shows the share of students who report to have worked in cocoa plantations in the last month for one hour or more at baseline, in response to the following question: "In the last month, have you engaged in one or more of the following activities, for one hour or more? Work in a cocoa plantation", as described in Table S1. The first bar comprises the whole sample in [15]; additional bars consider the indicated sub-samples. "Poorest" comprises households with monthly income reported by parents below 10,000 CFA ($\sim$ 19 USD), while "Richest" comprises those with monthly income reported by parents above 200,000 CFA ($\sim$ 372 USD). Rural and urban areas are defined according to parents' main occupation (agricultural or plantation activities are assigned to the former). Samples sizes are the following: (i) Total: 2,475; (ii) CP2: 1,285; (iii) CE2: 1,190; (iv) Boys: 1,237; (v) Girls: 1,238; (vi) Poorest: 139; (vii) Richest: 163; (viii) Urban: 1,234; (ix) Rural: 1,237.

Figure S8: Baseline correlation between child labor and educational outcomes

Panel A: Correlation between test scores and child labor

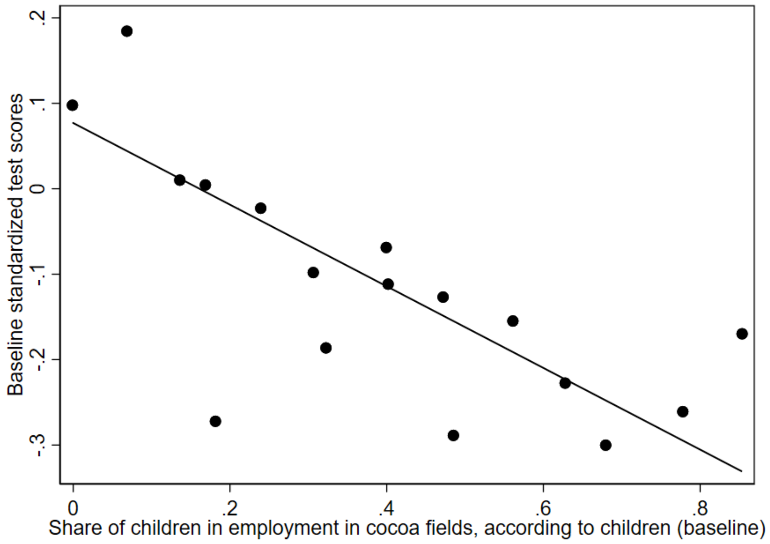

Panel B: Correlation between student dropouts and child labor

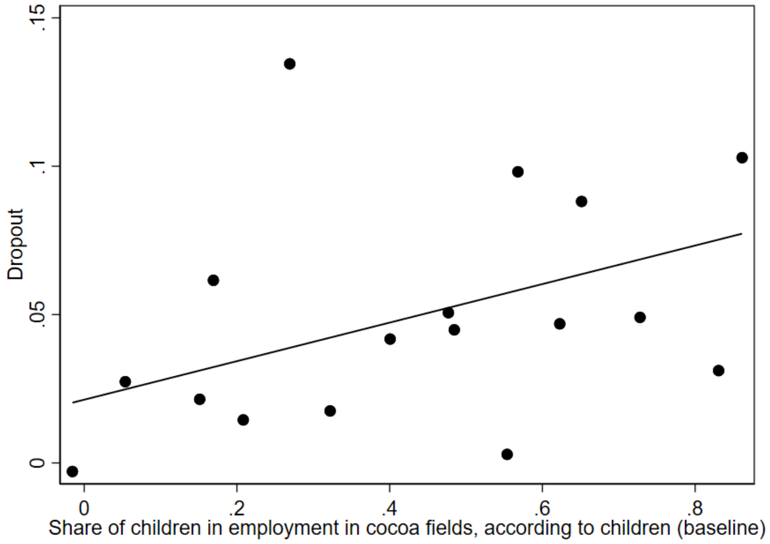

Notes: Panel A reports a bin-scatter plot of baseline standardized test scores as a function of baseline children in employment in cocoa fields (self-reported by children). Standardized test scores are a summary measure of numeracy and literacy test scores (averaging across each component, normalized by their mean and standard deviation in the control group), following [17]. Children in employment stands for the baseline share of students who report to have worked in cocoa plantations in the last month for one hour or more, in response to the following question: "In the last month, have you engaged in one or more of the following activities, for one hour or more? Work in a cocoa plantation", as described in Table S1. Test scores and the prevalence of children in employment are averaged at the classroom level. Panel B reports a bin-scatter plot of student dropout rates, based on administrative data (see [15]), as a function of baseline children in employment in cocoa fields (self-reported by children). Student dropouts and the prevalence of child labor are averaged at classroom level. Because student dropouts are defined at the end line, Panel B restricts observations to the control group of the intervention.

Figure S9: Histogram of the distance from households to the school (in km)

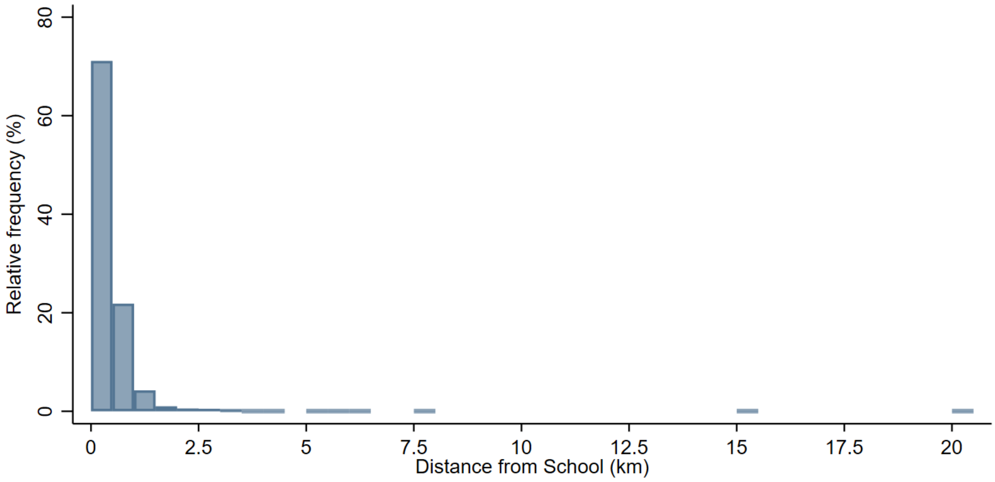

Notes: Distribution for the sub-sample of children with valid GPS coordinates associate with their household location.

Figure S10: Share of children in employment, by activity and reporting source

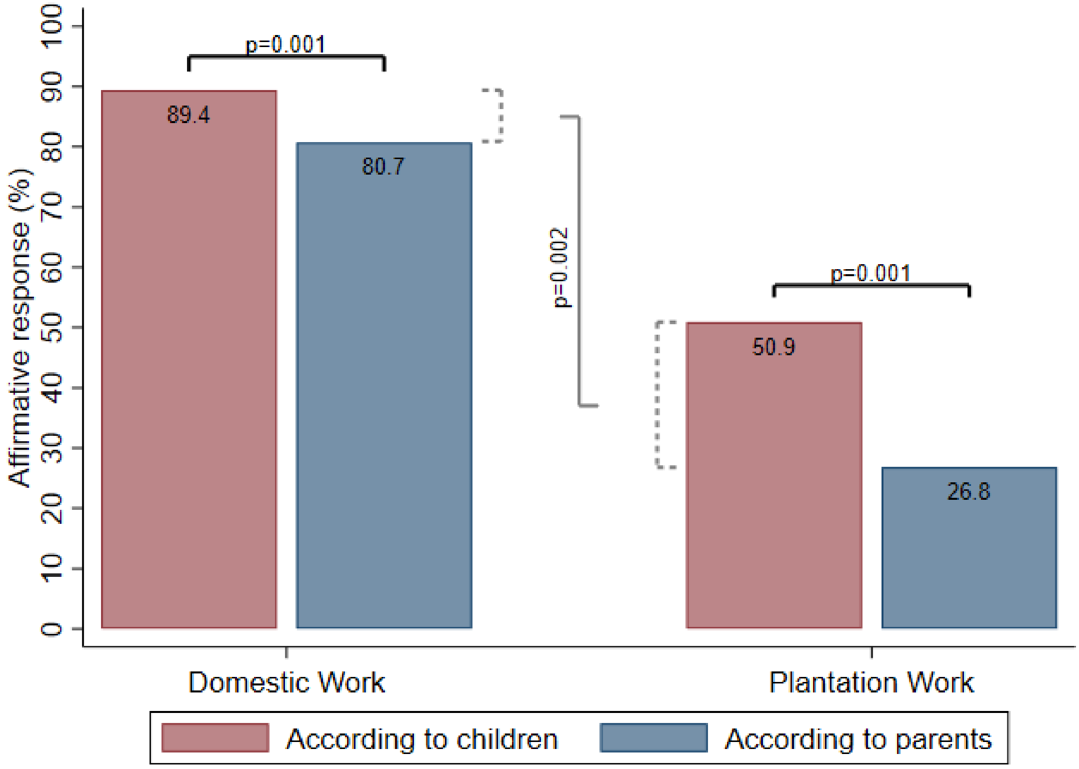

Notes: Bar shows the share of respondents that had an affirmative response to the works question in the endline survey. Specifically, parents were asked "In the last month, has any of your children engaged in one or more of the following activities, for one hour or more? Domestic work, such as buying food or cooking, cleaning the house, do the laundry, take care of children or other sick or old relatives?" and "Work in a cocoa plantation". Children were asked "In the last month, have you engaged in one or more of the following activities, for one hour or more? Domestic work, such as buying food or cooking, cleaning the house, do the laundry, take care of children or other sick or old relatives?" and "Work in a cocoa plantation". The p-value in the figure is relative to the paired t-test clustering standard errors at the regional level. The vertical p-value is relative to the test on the differences in responses given between parents and children between working conditions. Sample sizes are the following: (i) Children 2500: (ii) Parents: 2418 for domestic work question and 2019 for plantation work.

Prevalence of children in employment and hazardous work conditions measured in the follow-up survey

This Appendix compiles results on children's work hours and work conditions based on the follow-up survey (conducted in October 2019). Figure S11 plots the share of affirmative answers to different questions about children in employment. Figure S12 adds to the comparisons in the main text what teachers report about each student in the follow-up survey, when they were asked to report whether each of them had worked in cocoa fields at any point over the course of the previous school year. As the figure shows, teachers' and parents' reports (the latter, collected at the end-line survey) are statistically identical (p=0.23), and significantly under-estimate the end-line prevalence of children in employment according to children ($p<0.001$ in each case).

Figure S11: Parents' reports on children in employment and hazardous work conditions at the follow-up wave (October 2019)

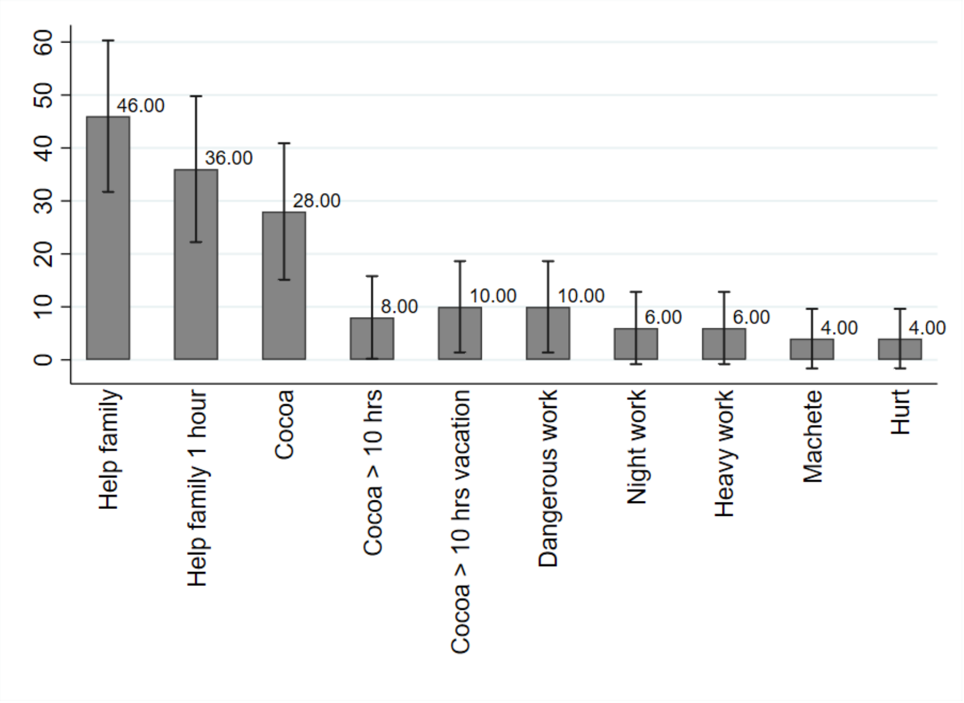

Notes: Bars show the share of students who parents have reported to be in the following conditions (yes/no questions): 1) Help family: "Did (child name) work at all to help you around home, assist in a family business or earn pocket money outside school hours under adult supervision during the school year last year?"; 2) Help family 1 hour: "Did (child name) work at all to help you around home, assist in a family business or earn pocket money outside school hours under adult supervision over 1 hour a week during the school year last year?"; 3) Cocoa: "Did (child name) work at all in cocoa fields during the school year last year?"; 4) Cocoa > 10 hrs: "Did (child name) work in cocoa fields more than 10 hours a week in a typical week last year?"; 5) Cocoa > 10 hrs vacation: "Did (child name) work in cocoa fields more than 10 hours a week during vacation last year?"; 6) Dangerous work: "Was (child name) involved in activities in cocoa fields such as clearing of forests and felling of trees, bush burning, manipulating agrochemicals or using sharp tools during the school year last year?"; 7) Night work: "Did (child name) work between 7 p.m. and 7 a.m. during the school year last year?"; 8) Heavy work: "Was (child name) engaged in heavy physical labor in a typical week last year?"; 9) Machete: "Did (child name) used a machete while woking in the fields last year?" 10) Hurt: "Did (child name) get hurt at least once while woking in the fields last year?". All measures were collected at the follow-up surveys [15]. Across all bars, the sample is restricted

to the control group.

Figure S12: Share of students who worked in cocoa plantations during the school year, according to different sources

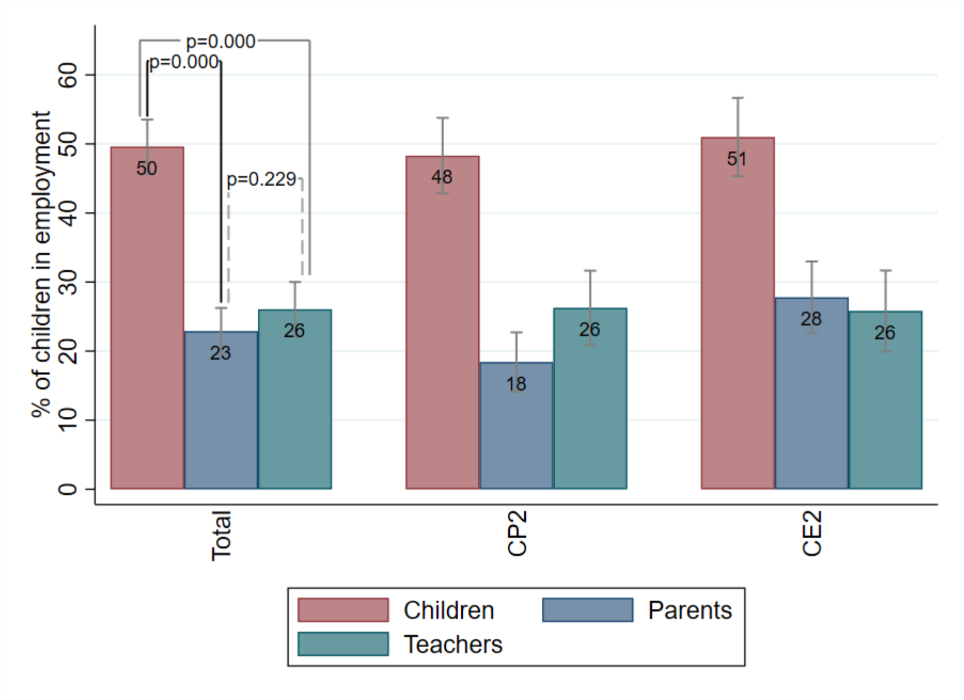

Notes: Bars show the share of students who have worked in cocoa plantations according to children (in red), parents (in blue), or teachers (in green). Children and parents answer at end line if the child engaged in one hour or more in cocoa plantation activities over the last month, as described in Table S1. Teachers answer at follow-up if each of their students worked in cocoa plantation at all over the last school year, as described in Table S1. Across all bars, the sample is restricted to the control group. The first set of bars comprises the whole sample in [15], while the additional ones split the sample by primary grades (CP2 and CE2). Sample sizes are the following: (i) Total: 625 ; (ii) CP2: 323; (iii) CE2: 302.

Additional results

This Appendix compiles additional results. Figures S13, S14 abd S15 complement our main results not only by breaking down our analyses of reporting discrepancies by region for which our surveys overlap with the certifier data, but also by expanding the sample to include additional households surveyed by ENVERITAS whose children were not necessarily all enrolled in school. The patterns we document are very robust across sub-samples and sample restrictions.

Next, Figure S16 illustrates how under-reporting patterns vary by urban status, at the classroom level. For almost all classrooms, adult reports are higher than children's self-reports (only 6 out of 198 observations are below the 45-degree line). The red line estimates a quadratic relation for mostly-rural classrooms (those with less than 20% of children living in urban areas), and the blue line, for mostly-urban classrooms (with over 80% of children living win urban areas). No mostly-urban classroom has a prevalence of children in employment greater than 20%. Within that lower range, however, under-reporting can be very high; for instance, the estimated relation predicts that if parents in these classrooms report a 10% prevalence of children in employment, the bias-adjusted prevalence is actually closer to 30%. For mostly-rural classrooms, under-reporting is predicted to be even the more striking the lower the prevalence in adult reports is. In these classrooms, a 10% reported prevalence would correspond to a nearly 45% bias-adjusted prevalence of children in employment. Naturally, in both cases, there is less room for under-reporting as prevalence according to adult reports increases. Next, Figure S17 documents the share of students reported to work over 10 hours a week at any point during the previous school year, according to both parents and teachers. Teachers were more conservative than parents when the question focused on employment during the school year, but less so if it focused on school holidays. Concretely, teachers identified child labor for only 2.8% of students during the school year (8%, according to parents) but for 12% of students during school holidays (10%, according to parents). Last, Figure S18 documents that, depending on reporting sources, the correlation between children in employment and educational outcomes at end line also systematically differs across treatment cells.

Figure S13: Validation of child labor measures using third-party data, households with all children in school

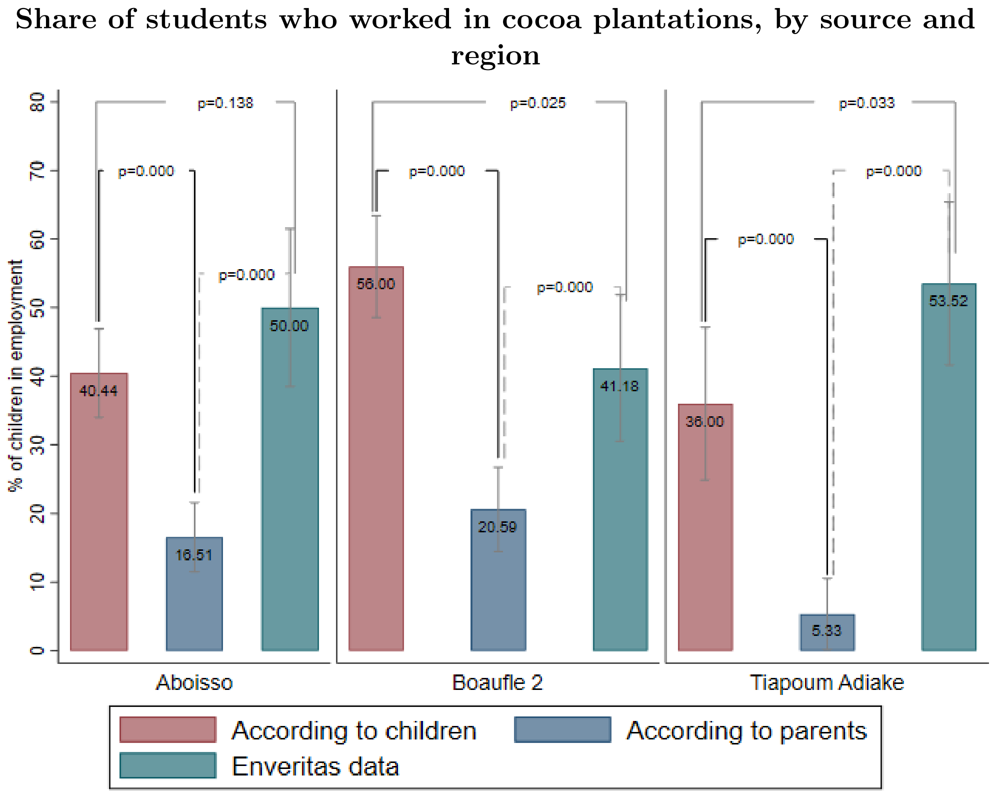

Notes: Columns show the share of children who worked at least an hour in cocoa fields over the previous month, according to children (in red), parents (in blue) and ENVERITAS (in green), along with 90% confidence bars. In the ENVERITAS data, we restrict attention to households whose *all* children between 6 to 11 years old were enrolled in school at the time of the survey. The figure reports the average prevalence for each region for which survey data overlaps with ENVERITAS data. Children answered the following question at end line: "In the last month, have you engaged in one or more of the following activities, for one hour or more? Work in a cocoa plantation", as described in Table S1. Parents answered the following question at end line: "I will now ask you some questions about activities that your children might have recently performed. In the last month, has any of your children engaged in one or more of the following activities, for one hour or more? Work in a cocoa plantation", as described in Table S1. In the survey conducted by ENVERITAS during the harvest season (identified through satellite imagery), farmers answered the following question: "Do any of your children between 6 and 16 years old help you work on the cocoa farm?". For the measures reported by children and parents, observations are restricted to the control group of the communication intervention. P-values from tests of proportions with unequal population variances (when children's or parents' reports are compared to ENVERITAS data; accounting for each source's intra-cluster correlation computed at the regional level), and from tests of proportions with equal population variances (when comparing children's and parents' reports) through Ordinary Least Squares regressions. Sample sizes are as follows: (i) Children: 475; (ii) Parents: 475; and (iii) ENVERITAS: 340.

Figure S14: Validation of child labor measures using third-party data, households with at least some children in school

Panel A: Share of students who worked in cocoa plantations, by reporting source

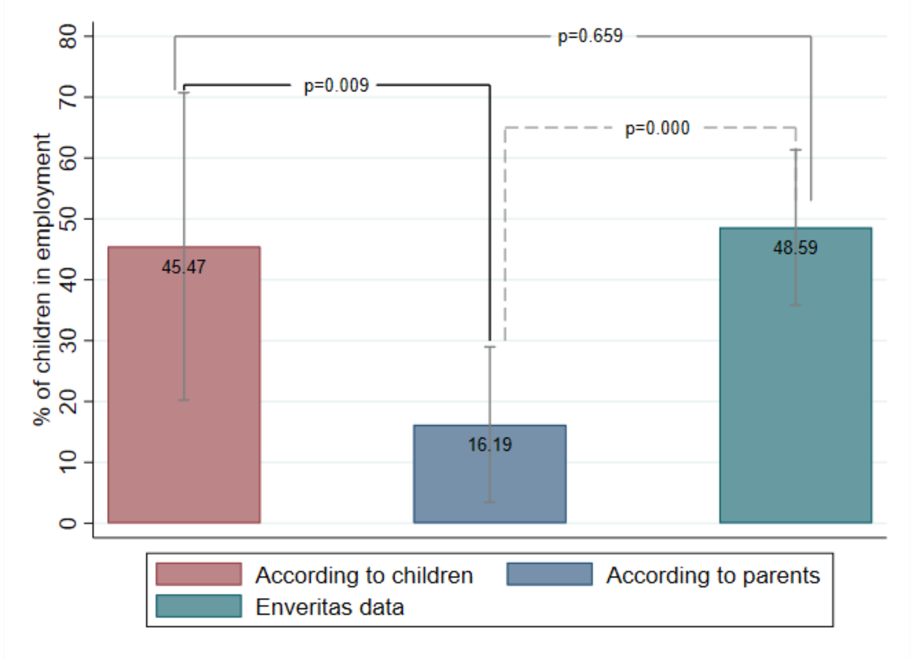

Panel B: Share of students who worked in cocoa plantations, by source and region

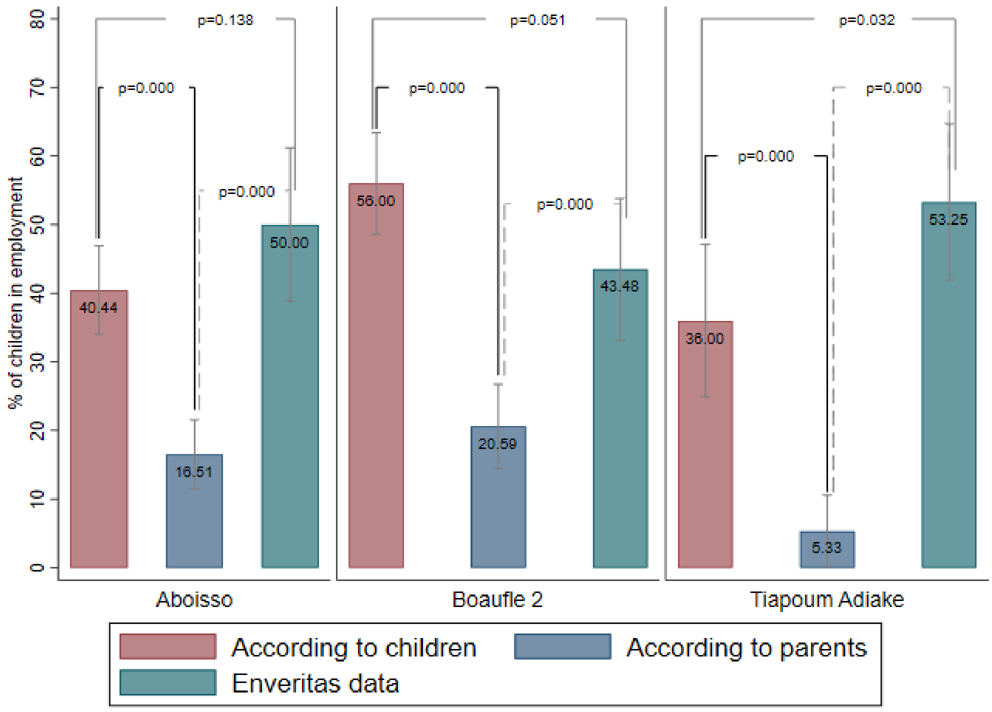

Notes: Columns show the share of children who worked at least an hour in cocoa fields over the previous month, according to children (in red), parents (in blue) and ENVERITAS (in green), along with 90% confidence bars. In the ENVERITAS data, we restrict attention to households with *at least one child*

between 6 to 11 years old enrolled in school at the time of the survey. Panel A reports the average prevalence across all regions for which survey data overlaps with ENVERITAS data. Panel B breaks down prevalence by region. Children answered the following question at end line: “In the last month, have you engaged in one or more of the following activities, for one hour or more? Work in a cocoa plantation”, as described in Table S1. Parents answered the following question at end line: “I will now ask you some questions about activities that your children might have recently performed. In the last month, has any of your children engaged in one or more of the following activities, for one hour or more? Work in a cocoa plantation”, as described in Table S1. In the survey conducted by ENVERITAS during the harvest season (identified through satellite imagery), farmers answered the following question: “Do any of your children between 6 and 16 years old help you work on the cocoa farm?”. For the measures reported by children and parents, observations are restricted to the control group of the communication intervention. P-values from tests of proportions with unequal population variances (when children’s or parents’ reports are compared to ENVERITAS data; accounting for each source’s intra-cluster correlation computed at the regional level, in Panel A), and from tests of proportions with equal population variances (when comparing children’s and parents’ reports) through Ordinary Least Squares regressions (clustering standard errors at regional level, in Panel A). Sample sizes are as follows: (i) Children: 475; (ii) Parents: 475; and (iii) ENVERITAS: 340.

Figure S15: Validation of child labor measures using third-party data, no sample restrictions

Panel A: Share of students who worked in cocoa plantations, by reporting source

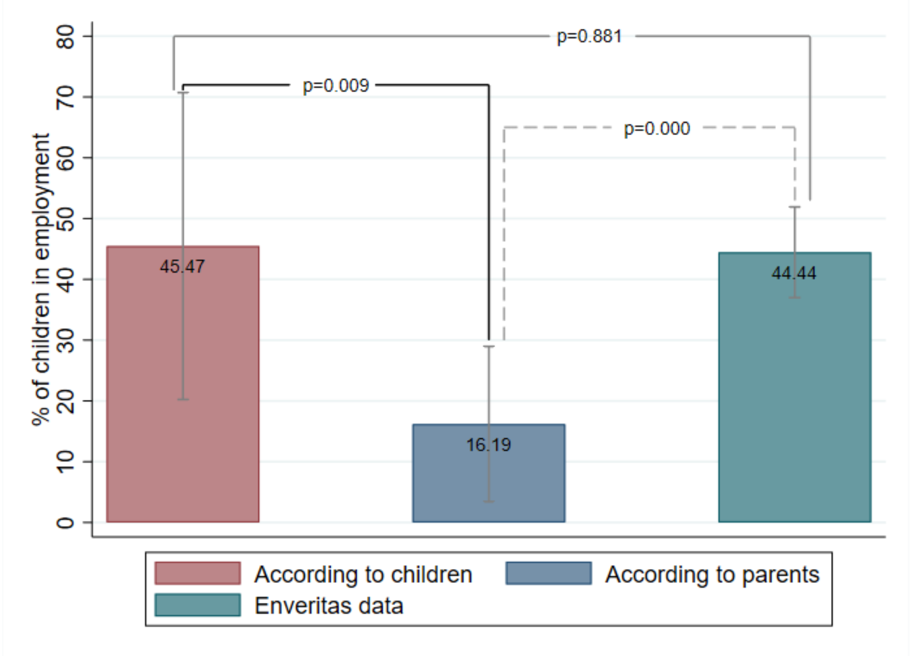

Panel B: Share of students who worked in cocoa plantations, by source and region

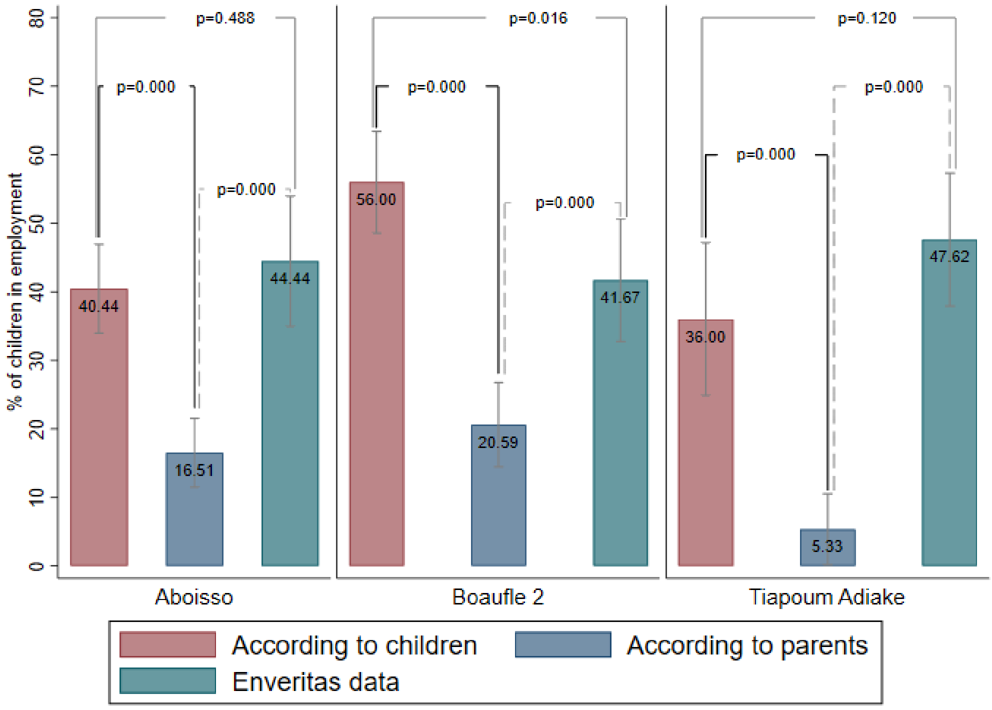

Notes: Columns show the share of children who worked at least an hour in cocoa fields over the previous month, according to children (in red), parents (in blue) and ENVERITAS (in green), along with 90% confidence bars. Panel A reports the average prevalence across all regions for which survey data overlaps

with ENVERITAS data. Panel B breaks down prevalence by region. Children answered the following question at end line: “In the last month, have you engaged in one or more of the following activities, for one hour or more? Work in a cocoa plantation”, as described in Table S1. Parents answered the following question at end line: “I will now ask you some questions about activities that your children might have recently performed. In the last month, has any of your children engaged in one or more of the following activities, for one hour or more? Work in a cocoa plantation”, as described in Table S1. In the survey conducted by ENVERITAS during the harvest season (identified through satellite imagery), farmers answered the following question: “Do any of your children between 6 and 16 years old help you work on the cocoa farm?”. For the measures reported by children and parents, observations are restricted to the control group of the communication intervention. P-values from tests of proportions with unequal population variances (when children’s or parents’ reports are compared to ENVERITAS data; accounting for each source’s intra-cluster correlation computed at the regional level, in Panel A), and from tests of proportions with equal population variances (when comparing children’s and parents’ reports) through Ordinary Least Squares regressions (clustering standard errors at regional level, in Panel A). Sample sizes are as follows: (i) Children: 475; (ii) Parents: 475; and (iii) ENVERITAS: 395.

Figure S16: Correlation between parents' and children's answer according to urbanization level

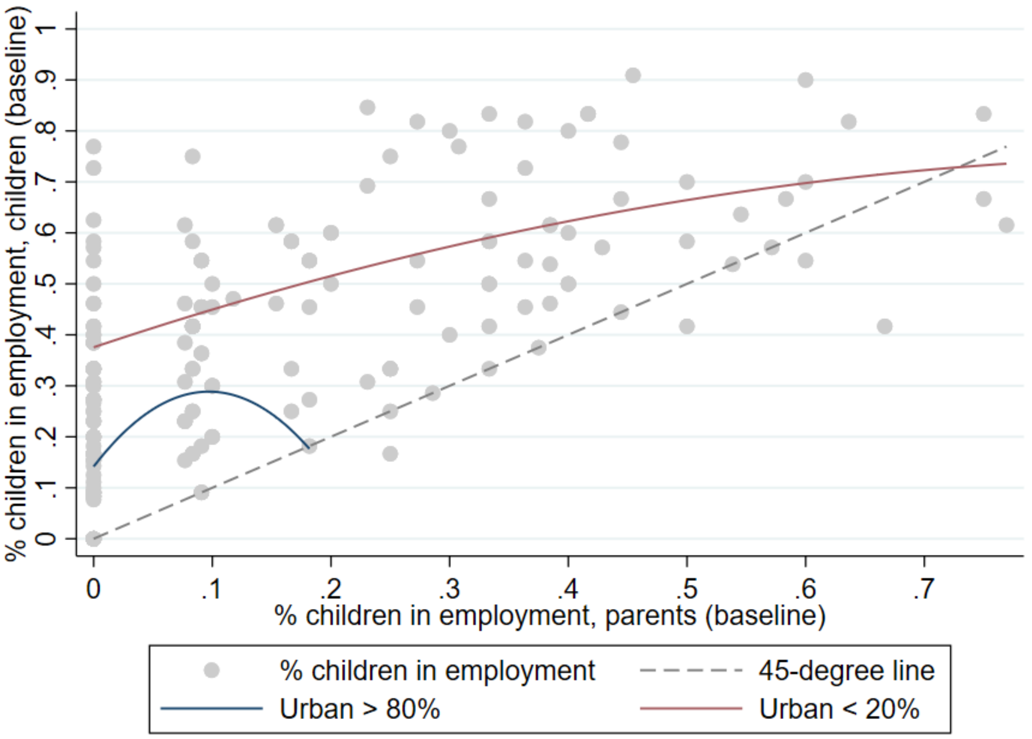

Notes: Data is aggregated at classroom level: average child labor according to children and parents were correlated according to composition on the classroom urbanization. "Urban > 80%" represents the quadratic fit between children's and parent's answer on child labor to classrooms that have more than 80% of their students in the urban category, while "Urban < 20%" represents the quadratic fit between children's and parent's answer on child labor to classrooms that have less than 20% of their students in the urban category, i.e., majority of students are from rural areas. Children answered the following question at baseline: "In the last month, have you engaged in one or more of the following activities, for one hour or more? Work in a cocoa plantation", as described in Table S1. Parents answered the following question at baseline: "I will now ask you some questions about activities that your children might have recently performed. In the last month, has any of your children engaged in one or more of the following activities, for one hour or more? Work in a cocoa plantation", as described in Table S1. Rural and urban areas are defined according to parents' main occupation (agricultural or plantation activities are assigned to the former).

Figure S17: Share of students who worked in cocoa plantations at least 10 hours/week, during the school year and during the school holidays

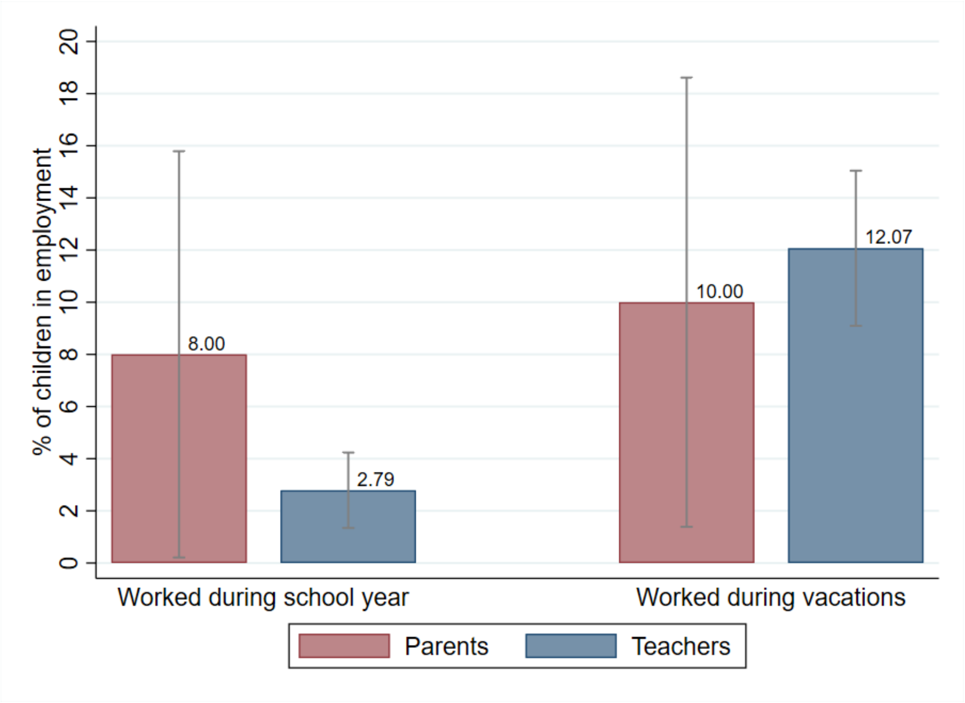

Notes: Bars show the share of students who worked in cocoa plantations during different periods and according to two different sources. Parents (in red) and teachers (in blue) answered whether the child worked 10 hours/week or more in cocoa fields during a typical week (LHS bars) and whether the child worked 10 hours/week or more in cocoa fields during school holidays in the previous school year, as described in Table S1. All measures were collected at the follow-up surveys [15]. Across all bars, the sample is restricted to the control group. Sample sizes are the following: (i) Parents: 200; and (ii) Teachers: 2,500.

Figure S18: Correlation between end-line test scores and child labor, by source and treatment status

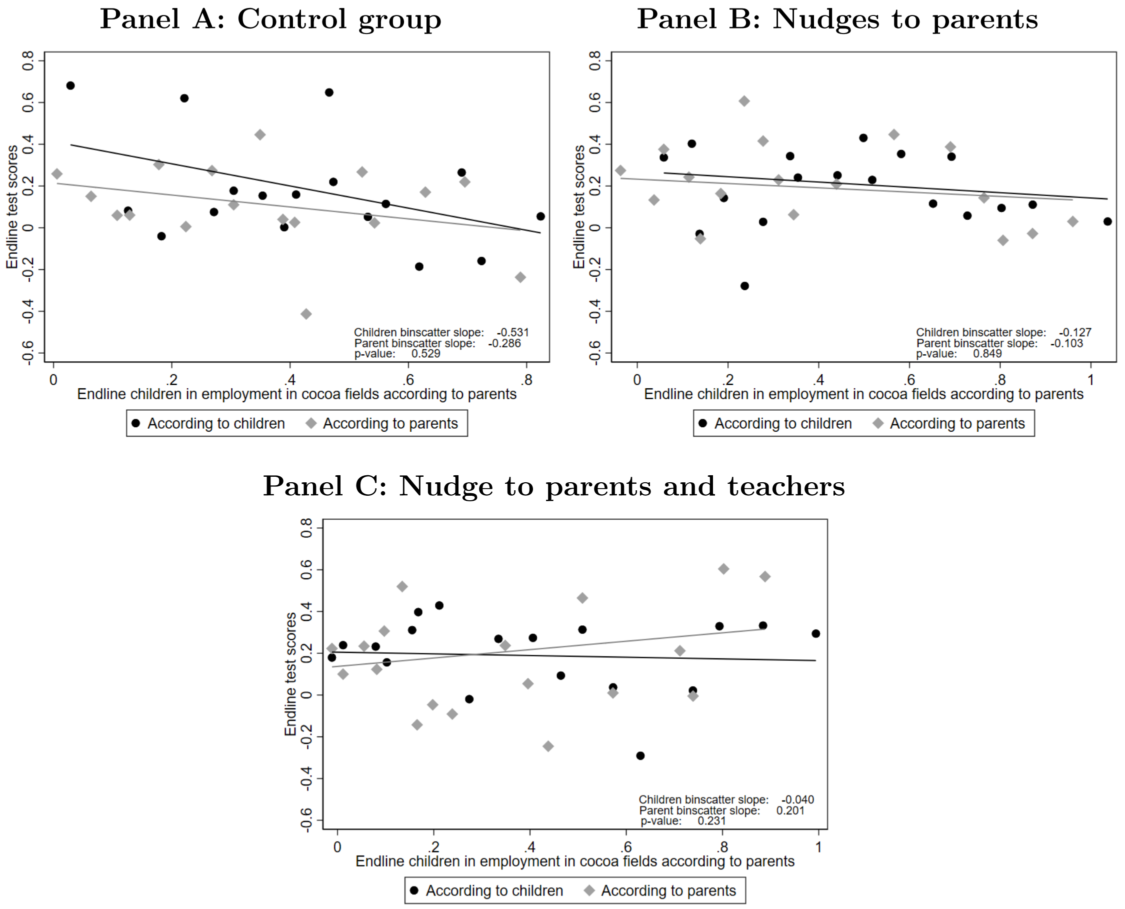

Notes: All panels show bin-scatter plots of end-line standardized test scores as a function of children in employment in cocoa fields at end line. Each panel estimates linear relation between the variables according to different measures of child labor; that based on children's self-reports is shown in black, and that based on parents' reports is shown in gray. Children answered the following question: "In the last month, have you engaged in one or more of the following activities, for one hour or more? Work in a cocoa plantation", as described in Table S1. Parents answered the following question: "I will now ask you some questions about activities that your children might have recently performed. In the last month, has any of your children engaged in one or more of the following activities, for one hour or more? Work in a cocoa plantation", as described in Table S1. Standardized test scores are a summary measure of numeracy and literacy test scores (averaging across each component, normalized by their mean and standard deviation in the control group), following [17]. Panel A restricts the sample to the control group of the intervention; Panel B, to parents who randomly assigned to nudges (via text or or audio messages) in schools where teachers were not assigned to the intervention; and Panel C, to parents assigned to nudges (via text or audio messages) in schools where teachers were also assigned to nudges (via text messages); see [15].

Table S3: Robustness of treatment effects of nudges by reporting source and household remoteness, winsorizing outlier distance values

	Children in employment (self-report)	Children in employment (parent's report)		
	(1)	(2)	(3)	(4)
**Panel A - Work in cocoa fields**				
Nudge to parents	0.054	0.089*	0.120**	0.135**
	(0.040)	(0.045)	(0.056)	(0.065)
Distance to school (km)				-0.009
				(0.035)
Nudge to parents × Distance				-0.037
				(0.058)
Sample mean	0.498	0.221	0.230	0.230
R-squared	0.115	0.091	0.094	0.095
Observations	2,246	2,176	1,608	1,608
**Panel B - Domestic work**				
Nudge to parents	0.046**	0.051**	0.066**	0.065**
	(0.020)	(0.022)	(0.026)	(0.032)
Distance to School (km)				-0.042
				(0.029)
Nudge to Parents × Distance				0.004
				(0.051)
Sample mean	0.878	0.789	0.794	0.794
R-squared	0.034	0.044	0.050	0.052
Observations	2,246	2,175	1,607	1,607
Controls (baseline survey)	Yes	Yes	Yes	Yes

Notes: In this specification, we winsorize outliers with respect of distance to school at the 99th percentile (where $distance_{99\text{th percent.}} \approx 3.88$), hence, outliers values are substituted by the distance value at the 99th percentile. In column (1), Children in employment (self-report) = 1 if the child answered affirmatively to the working question: "In the last month, have you engaged in one or more of the following activities, for one hour or more? Work in a cocoa plantation" in Panel A, "Domestic work, such as buying food or cooking, cleaning the house, do the laundry, take care of children or other sick or old relatives", in Panel B. In columns (2) to (4), Children in employment (parent's report) = 1 if the parent answered affirmatively to the question "In the last month, has any of your children engaged in one or more of the following activities, for one hour or more? Work in a cocoa plantation", in Panel A, and "Domestic work, such as buying food or cooking, cleaning the house, do the laundry, take care of children or other sick or old relatives", in Panel B. Distance from school (km) is the linear distance between the home and their child's school GPS locations, in kilometers; Nudge to parents = 1 if the parent (but not teachers) was assigned to nudges, and 0 otherwise. Baseline controls include treatments arms, child gender, grade indicators, standardized test scores (averaged across numeracy and literacy); and summary measures of parental engagement, student effort, socio-emotional skills, working memory, visual attention, impulsivity, self-esteem, and mindset (see [14]. Summary measures computed following [17], standardizing each component by normalizing values by the mean and standard deviation of the control group at the baseline survey within each grade, and then averaging over all standardized components. In columns (3) and (4), sample restricted to observations with valid home GPS coordinates. All regressions estimated through Ordinary Least Squares with standard errors clustered at the classroom level.
*** p<0.01, ** p<0.05, * p<0.10

Predicting the bias-adjusted prevalence of child labor

Informed by the statistical equivalence between ENVERITAS data (treated as the ground truth) and children's reports of the prevalence of children in employment, we can estimate the relation between adult reports (the ILO standard measure) and children's self-reports not only to compute the extent of under-reporting in our data, but also to predict what accurate reports would have been in geographical units without ENVERITAS data. In this Appendix, we show how estimating this *bias-adjustment factor* for children in employment allows us to compute the bias-adjusted prevalence of child labor for Côte d'Ivoire and Ghana, under the assumption that this relation is the same conditional on the local characteristics we can measure. We focus on these two countries because 2/3 of the global cocoa production originates within their borders and because our surveys asked about children in employment specifically in cocoa farms.

According to the ILO definition:

$$CL_i = CE_i \text{ x } HC_i, \tag{1}$$

where CL_i is the share of child labor in country i, CE_i is the share of children in employment in country i, and HC_i is the share of children in employment in country i who work in hazardous conditions (= 1 for children under 12, and = % of those working long hours and/or under heavy or dangerous work conditions, otherwise).

Once we determine that children's self-reports are accurate and, as such, can be used as the ground truth, in our data we observe both CE_j, the share of children in employment reported by parents in classroom j, and CE_j^*, the ground truth for children in employment in classroom j. We are interested in predicting CE_i^* for all countries $i \in I$. To do that, we first estimate:

$$CE_j^* = f(CE_j, X_j) + \varepsilon_j, \tag{2}$$

where $f(CE_j, X_j)$ is a function of children in employment reported by parents in classroom j and other classroom characteristics X_j, and ε_j is an error term.

We then use our estimates to compute:

$$\hat{CE}_i^* = \hat{\alpha}_i \text{ x } CE_i, \tag{3}$$

where $\hat{\alpha}_i = \frac{\hat{f}(CE_i, X_i)}{CE_i} \geq 1$.

Those same estimates can be used to recover CL_i^*, the ground truth for child labor in country i. This is thanks to the fact that HC_i is free of bias (since = 1 for 5-11 year olds, and elicited directly from 12-14 year-old children), and to the multiplicative nature of its definition:

$$CL_i^* = CE_i^* \text{ x } HC_i = \hat{\alpha}_i \text{ x } CE_i \text{ x } HC_i = \hat{\alpha}_i \text{ x } CL_i \tag{4}$$

Concretely, we estimate a quadratic polynomial, allowing the extent of under-reporting to vary with both the local level of children in employment reported by parents and with the local urbanization rate:

$$CE_j^c = \sum_{k=0}^{2} \beta_k \left(CE_j^p\right)^k + \sum_{k=0}^{2} \gamma_k \left[\left(CE_j^p\right)^k \text{ x } urban_j\right] + \varepsilon_j, \tag{5}$$

where CE_j^c is % child labor according to children in classroom j, CE_j^p, that according to parents in classroom j, and $urban_j$ is the % of students from urban areas in classroom j.

We only estimate heterogeneity with respect to children in employment reported by adults and urbanization rates because these are available both in our data and in the World Development Indicators. Other variables, like annual per capita income, are not available in our data.

Last, we compute $\hat{\alpha}_i = \max\left\{1, \frac{\hat{CE}^c_i}{CE_i}\right\}$, where $\hat{CE^c}_i$ is the predicted value of CE^c for country i using equation (5). As the formula indicates, we constrain the estimated bias-adjustment factor (as well as its confidence interval) to be greater or equal to 1. In practice, this constraint does not affect Côte d'Ivoire or Ghana.

World Development Indicators track child labor for 97 countries (those where the issue is considered to be relevant by the data collection organizations, such as UNICEF country offices), focusing on 7-14 year-old children. With the bias-adjustment factor that we predict for Côte d'Ivoire and Ghana using the procedure described above, we compute country-level bias-adjusted prevalence of child labor and number of child workers (and accompanying 95% CIs) using data from the most recent year available for each country in the World Development Indicators. To arrive at the bias-adjusted prevalence figures, we have to rely, in addition, on World Development Indicators data on the number of 0-14 year old children by country, and subtract these from the UNICEF figures for under-5 children by country. For both population counts, we use 2020 data – assuming that child labor indicators remained constant since their most recent measurement.

Table S4: Relation between children's and parents' reports at the classroom-level

	(1)
Child labor according to parents	0.463
	(0.294)
Urban	-0.361***
	(0.056)
(Child labor according to parents)^2	-0.069
	(0.439)
(Child labor according to parents)*Urban	0.850
	(0.633)
(Child labor according to parents)^2*Urban	-1.491
	(1.270)
Constant	0.477***
	(0.041)
Observations	198
Adjusted R^2	0.570

*Notes: * p<0.10, **p<0.05, ***p<0.01.*
Estimation based on the following equation: $CL_c = \alpha + \beta_1 CL_p + \beta_2 Urban + \beta_3 {CL_p}^2 + \beta_4 CL_p * Urban + \beta_5 {CL_p}^2 * Urban + \varepsilon$, in which: CL_c is child labor according to children, CL_p is child labor according to parents, and $Urban$ the percentage of students living in urban areas. Results are for classroom-level averages. Rural and urban areas are defined according to parents' main occupation (agricultural or plantation activities are assigned to the former). Child labor according to parents corresponds to the answer on the following question "I will now ask you some questions about activities that your children might have recently performed. In the last month, has any of your children engaged in one or more of the following activities, for one hour or more? Work in a cocoa plantation", as described in Table S1. Child labor according to children stands for the baseline share of students who report to have worked in cocoa plantations in the last month for one hour or more, in response to the following question: "In the last month, have you engaged in one or more of the following activities, for one hour or more? Work in a cocoa plantation", as described in Table S1. All variables correspond to baseline answers.

Table S5: Bias-adjusted prevalence of child labor by country

Country	Prevalence			Children in employment		
	**WDI***	**Bias-adjusted**	**95% CI**	**WDI****	**Bias-adjusted**	**95% CI**
Côte D'Ivoire	36.50%	50.75%	[44.80%; 56.71%]	2,488,650	3,460,540	[3,054,542; 3,866,538]
Ghana	28.80%	46.67%	[40.74%; 52.61%]	2,122,198	3,439,275	[3,001,906; 3,876,644]

Notes: * using the latest available WDI figures, following [18]; ** using WDI and UNICEF population figures for 0-14 and under-5 children for 2020. Bias-adjusted prevalence of child labor based on Table S4's estimates for country-specific adjustment factors.
