# Supplementary material for: Measuring Child Labor: The Who’s, the Where’s, the When’s, and the Why’s
Source: PLoS One. 2025 Jun 9;20(6):e0322987. doi: 10.1371/journal.pone.0322987 (PMC12148184; doi:10.1371/journal.pone.0322987)
Supplement: Attachment — Submitted filename: Rebuttal Letter.pdf [file pone.0322987.s003.pdf]

Stanford | GRADUATE SCHOOL OF EDUCATION

520 Galvez Street – CERAS 519
Palo Alto, CA, 94305
United States

March 4, 2025

Dear Dr. Ekwebelem,

Thank you very much for the thoughtful feedback on our original submission, and for the opportunity to revise our work for PLOS ONE. We have now carefully revised the manuscript to follow all style requirements of the journal, and to incorporate all comments by the referees.

In particular, we have conducted additional empirical exercises to ensure that our findings are robust to (1) removing outlier observations, and (2) parametric assumptions of our econometric specification.

We have also carefully rewritten large segments of the paper, unifying references to different child labor statistics to avoid confusion, and more carefully characterizing previous findings from the literature in the Conclusions.

All in all, we believe that we have successfully addressed all concerns raised by the referees and by the Editor and that the manuscript is much stronger as a result.

Thank you once again for the attention dedicated to our submission, and for your numerous inputs – which have helped us significantly improve the paper.

We include a point-by-point response to the referees comments below (in **blue**).

Stanford | GRADUATE SCHOOL OF EDUCATION

520 Galvez Street – CERAS 519
Palo Alto, CA, 94305
United States

Reviewer #1: Hi Dear Author,
An important subject has been touched upon and it is a beautiful work in terms of design. The suggestion should be refocused and stated more clearly. Explain the practical implication and further research suggestions based on the finding.

Thank you for the encouraging feedback and for the suggestion to make the practical implications of our findings and the directions for future research more clear.

We have compiled below that changes we have made accordingly in the Conclusions section, as follows:

> "Data on the prevalence of child labor across space and over time is critical for governments and international organizations committed to ensuring children's rights. In the cocoa industry, particularly intensive in child labor given its low rate of mechanization, data issues have posed important challenges to monitoring and enforcement efforts by international organizations and policymakers over the years.
>
> Our finding that child labor statistics following the ILO methodology not only misrepresent the prevalence of child labor, but also mischaracterize its trends (where interventions were in place), raises critical concerns. While the general sentiment of the literature on child labor is that substantial progress has been achieved in recent decades ('*[a]n important lesson from all the literature reviewed herein is that child labor can change dramatically and quickly in countries as a result of changes in the economic and policy environment*'; Edmonds and Theoharides, 2021, pp. 27-28), our results call that sentiment into question Concretely, Appendix F in Supplementary Materials uses our estimates to calibrate predictions of the bias-adjusted prevalence of child labor in Côte d'Ivoire and Ghana. Under the stated assumptions, child labor could affect nearly 6.9 million children in these countries, 50% more than in the latest World Development Indicators' statistics. Naturally, this does not invalidate the claim that child labor might be caused by lack of economic development or that it can respond to policy changes (Edmonds and Theoharides, 2021); however, it does raise caution about carefully interpreting changes in adult reports in contexts of fast transitions – in light of how such changes might impact not only the economic fundamentals that generate trade-offs between employing children vis-a-vis investing in their human capital, but also, reporting biases themselves (e.g., by changing parents' beliefs about what is socially appropriate).
>
> Based on our results, asking children independently about whether they work (and, if so, how many hours) could yield child labor indicators consistent with costly-verification data. There are, however, technical challenges involved in interviewing children, including whether children are asked inside or outside the household, and variations in the understanding of what exactly characterizes 'work' by children in different countries (see Guarcello et al., 2010 and Dillon, 2010 for a broader discussion of different framing issues). While the experiences of NORC and Tulane University in Côte d'Ivoire and Ghana since 2008-09 could inform the replication of self-reports elicitation of child labor by other agencies moving forward, surveying children directly involves some ethical challenges as

Stanford | GRADUATE SCHOOL OF EDUCATION

520 Galvez Street – CERAS 519
Palo Alto, CA, 94305
United States

well. In particular, children might be exposed to violence if interviews trigger backlash from parents, even when they are conducted in a different setting (e.g., schools). These concerns also limit the potential of technologies worn by children, such as GPS trackers, despite their validated accuracy (Dillon et al., 2017).

For those reasons, finding ways to limit reporting biases in adult surveys may be a superior (and necessary) alternative. Progress in this space has, however, been slow. Most studies focus on indirect elicitation methods (i.e., list experiments, in various forms), but these methods generate estimates that are typically imprecise and not necessarily closer to the 'ground truth'. In contrast, leveraging technologies to survey adults in communities less subject to previous sensitization and during periods when it is easier to observe child labor might be a much better way forward. Naturally, that also involves its own challenges. Although we have documented that remoteness is not systematically associated with the true prevalence of child labor (based on children's self-reports) in the close vicinity of schools, it might be that the communities surveyed based on satellite imagery are not representative of the territories or the populations of interest, leading to biased aggregate estimates of children in employment and child labor. Moreover, if remote sensing through satellite imagery is imperfect (e.g., if the geographical coverage of the available data is selective either when it comes to which regions it covers or which farms it surveys within each region), then the estimates will again be biased. There are also cost considerations. Obtaining access to high-frequency data that can inform prediction models (and hiring staff or external vendors to train and update such models) might be expensive. Such requirements might limit the ability to learn timely about how child labor evolves across space, particularly in response to policy interventions.

While we expect our contributions to generalize beyond the Ivorian cocoa industry, a limitation is that our sample consists entirely of school children – all of whom were enrolled at the time of our surveys. This excludes children who had dropped out of school (or never enrolled in the first place), presumably more likely to work in cocoa fields. While their parents might be less sensitive to social desirability biases (since their children are not in school), there are other potential sources of social pressure to under-report children in employment – particularly economic pressures linked to restrictions to child labor in global supply chains. Investigating if parents' under-reporting changes with children's school enrollment remains a promising avenue for future work".

Thank you once again for your inputs, which have helped us greatly improve the paper.

Stanford | GRADUATE SCHOOL OF EDUCATION

520 Galvez Street – CERAS 519
Palo Alto, CA, 94305
United States

Reviewer #2: The topic is good, but needs some follow-up:

1. In the introduction, it is necessary to dig deeper into previous studies on child labor in terms of the development of measurement methods, child labor trends, the role of parents in increasing or reducing child labor and the success or failure of the government's role in supervising child labor, as well as the influence of remote and non-remote areas on child labor trend. These things can be demonstrated by providing examples of comparisons with child labor levels in other countries in various types of industry that have the potential to use child labor.

Thank you for the encouraging feedback and for the suggestion to more deeply characterize the previous literature on measuring methodologies, and child labor levels and trends.

We discuss different measurement methodologies in detail, both in the Introduction and in the Conclusions section. In the former:

> "Previous studies were not able to document the extent of under-reporting by parents. These papers rely on three types of comparisons. First, comparisons between parents' reports under different conditions for social desirability bias (e.g., Jouvin, 2021), to document that such biases exist – but unable to pin down their magnitude. Second, comparisons between adults' and children's reports (Dillon, 2010; Galdo et al., 2020), to document that discrepancies exist – but unable to pin down what the 'ground truth' is. Third, comparisons between an objective measure of children in employment and children's self-reports (Dillon et al., 2017), to document that the latter are accurate – but unable to pin down under-reporting by parents. Similarly to ours, the latter study documents that children accurately report the number of hours they work, using logs from GPS trackers worn by different household members to verify data from surveys and activity diaries. The study, however, actively refrained from having enumerators ask adults about child labor. In the absence of parents' reports, it could not document whether parents under-report children in employment and, if so, by how much."

In the Conclusions section, we complement this characterization as follows:

> "Based on our results, asking children independently about whether they work (and, if so, how many hours) could yield child labor indicators consistent with costly-verification data. There are, however, technical challenges involved in interviewing children, including whether children are asked inside or outside the household, and variations in the understanding of what exactly characterizes 'work' by children in different countries (see Guarcello et al., 2010 and Dillon, 2010 for a broader discussion of different framing issues). While the experiences of NORC and Tulane University in Côte d'Ivoire and Ghana since 2008-09 could inform the replication of self-reports elicitation of child labor by other agencies moving forward, surveying children directly involves some ethical challenges as well. In particular, children might be exposed to violence if interviews trigger backlash from parents, even when they are conducted in a different setting (e.g., schools). These concerns also limit the potential of technologies worn by children, such as GPS trackers, despite their validated accuracy (Dillon et al., 2017).

Stanford | GRADUATE SCHOOL OF EDUCATION

520 Galvez Street – CERAS 519
Palo Alto, CA, 94305
United States

> For those reasons, finding ways to limit reporting biases in adult surveys may be a superior (and necessary) alternative. Progress in this space has, however, been slow. Most studies focus on indirect elicitation methods (i.e., list experiments, in various forms), but these methods generate estimates that are typically imprecise and not necessarily closer to the 'ground truth'. In contrast, leveraging technologies to survey adults in communities less subject to previous sensitization and during periods when it is easier to observe child labor might be a much better way forward. […]"

When it comes to child labor levels and trends, we agree that the previous version of the manuscript introduced some confusion due to the multiplicity of measures mentioned in the introduction. We have now re-written the Introduction to remove ambiguity, as follows:

> "Child labor is a pervasive practice in agriculture, especially in West Africa, where the global cocoa industry sources roughly half of its produce. Defined according to international standards and national legislation, child labor typically characterizes any work by children under the age of 12, and excessive or risky work by 12-17 year-olds (see Section 2.1.1). 2020 data from the International Labor Organization (ILO) documented 160 million child workers worldwide – 9.6% of all 5-17 year-old children (ILO and UNICEF, 2021). Strikingly, that figure was over 2-fold in Sub-Saharan Africa (23.9%). In Côte d'Ivoire, the setting of our study, 15% of the cocoa industry employees that year were children (Sadhu et al., 2020)."

To avoid confusion while still being able to reference commonly cited statistics on child labor levels and trends – albeit measured in different geographies and using different methodologies –, we moved those descriptions to a new subsection within Methods and materials ('How child labor is measured'), as follows:

> "There are striking differences between children's and adults' reports in the few settings where both have been asked independently about children in employment. According to NORC (a research institute based at the University of Chicago), which surveys children directly about the number of hours worked in cocoa fields, 38% of 5-17 year olds in Côte d'Ivoire reported to have worked in 2018-19 (Sadhu et al., 2020); in contrast, the ILO figure for 2016 – based on adult reports for children employment – was only 23% (ILO and UNICEF, 2021). Even worse, different sources tell very different stories about child labor's recent trends. While ILO data indicate a 38% decrease in child labor worldwide since 2000 (Christian Science Monitor, 2019), NORC data record a nearly 65% increase in child labor since 2008-09 (Sadhu et al., 2020).
>
> While these differences are suggestive that official statistics based on adult reports are biased, it is hard to be sure; besides differences in whom is surveyed, discrepancies across surveys might also accrue to differences in their geographical coverage or in the timing of data collection, or to other methodological differences. Moreover, in the absence of verification, it is unclear whether children's self-reports do not suffer from reporting biases too."

520 Galvez Street – CERAS 519
Palo Alto, CA, 94305
United States

While it is hard to comment on government's success or failures in supervising child labor (in remote areas or otherwise) given such data reliability issues, we now added a more explicit take in the Conclusions section, as follows:

> "Our finding that child labor statistics following the ILO methodology not only misrepresent the prevalence of child labor, but also mischaracterize its trends (where interventions were in place), raises critical concerns. While the general sentiment of the literature on child labor is that substantial progress has been achieved in recent decades ('*[a]n important lesson from all the literature reviewed herein is that child labor can change dramatically and quickly in countries as a result of changes in the economic and policy environment*'; Edmonds and Theoharides, 2021, pp. 27-28), our results call that sentiment into question Concretely, Appendix F in Supplementary Materials uses our estimates to calibrate predictions of the bias-adjusted prevalence of child labor in Côte d'Ivoire and Ghana. Under the stated assumptions, child labor could affect nearly 6.9 million children in these countries, 50% more than in the latest World Development Indicators' statistics. Naturally, this does not invalidate the claim that child labor might be caused by lack of economic development or that it can respond to policy changes (Edmonds and Theoharides, 2021); however, it does raise caution about carefully interpreting changes in adult reports in contexts of fast transitions – in light of how such changes might impact not only the economic fundamentals that generate trade-offs between employing children vis-a-vis investing in their human capital, but also, reporting biases themselves (e.g., by changing parents' beliefs about what is socially appropriate)."

2. For discussion, it is necessary to add an explanation of the results of the correlation between child labor and test scores and drop outs that shown in the supplementary data. Then, also need to explain the bias in the reporting of parents, children and Enveritas. All three show different results, so it is necessary to explain which one is more accurate. If the researcher suggest that Enveritas can give the better data that other instrument, so please explain and give the evidence that Enveritas can lower the Bias result of Child labor report. Apart from that, there is also a need for discussion supported by literature regarding the finding that the more remote the area, the less child labor. Also, why child labor is more often found for domestic work than plantation work. Then conceptualize the findings in Cote D'Ivore in general regarding the development of child labor globally.

We describe the correlation between child labor, test scores and school dropouts in Section 3.1 ('Descriptive statistics based on children's self-reports'), as follows:

> "Appendix C also illustrates correlations between classroom-level prevalence of child labor and educational outcomes. Consistent with common sense, classrooms with a higher share of children in employment at baseline feature lower test scores by the beginning of the school year, and higher dropout rates over the course of the school year. Estimating a linear relation between the variables in each case suggests that moving from 0% to 20% children in employment is associated with about 0.08 s.d. lower test scores – what children tend to learn in one school quarter, and the magnitude of effect sizes of many educational

Stanford | GRADUATE SCHOOL OF EDUCATION

520 Galvez Street – CERAS 519
Palo Alto, CA, 94305
United States

> interventions, such as nudges to parents evaluated in this setting (Lichand and Wolf, 2022). Similarly, moving from 0% to 40% children in employment is associated with roughly doubling dropout rates.
>
> While these associations are not causal, they help understand the centrality of the issue for governments and international organizations monitoring children's rights. This is why accurate measurement is key."

In explaining parents' biases, we mention in the Introduction:

> "While such figures have mobilized massive global attention and resources to combat child labor, a key challenge in interpreting them is reliability. This is because while official statistics on child labor are essentially based on adult reports (see Section 2.1.1), there are many reasons why parents may fail to truthfully disclose children in employment. They might fear that admitting to child labor (e.g., that their under-12 year-old children help during the harvest season) could lead them to face legal action from child protective services, or adversely impact their livelihoods (e.g., if the companies sourcing from them are punished for child labor in their supply chain, which could trickle down to lower prices or even to discontinuation of their income; see Lépine et al., 2022). Importantly, if parents omit children in employment, child labor will be under-estimated in the data – since child labor is *contained* in children in employment."

Next, when it comes to assessing the relative accuracy of different data sources, in Section 2.4.1 ('Assessing the certifier claim that remoteness is inversely related to reporting biases'), we state:

> "To assess whether remoteness is indeed inversely related to reporting biases, we estimate how children's and parents' reports vary with their household's distance to the school (in km). […]
>
> Taking advantage of natural variation in distance to the school, we estimate a linear model for the association between distance and children's reports, and between the former and the discrepancy between children's and parents' reports. […]

Then, in Section 3.2 ('Household remoteness and reporting discrepancies'), we add (emphasis in **bold**):

> "**If remote communities are indeed less often or less intensely sensitized**, a challenge is that remoteness might affect both under-reporting and the true prevalence of child labor. To separate these potential effects, Table 1 starts by assessing whether children's self-reports vary systematically with our measure of remoteness (in column 1); and columns (2) to (4) assess how the discrepancy between children's and parents' reports varies with that measure. Panel A focuses on labor in cocoa fields; and Panel B turns to domestic labor. All columns control for children's self-reports; column (3) further controls for the school-

Stanford GRADUATE SCHOOL OF EDUCATION

520 Galvez Street – CERAS 519
Palo Alto, CA, 94305
United States

> level prevalence of child labor (according to children) and its interaction with each child's self-report, as those might also affect the extent of reporting biases; column (4) additionally controls for baseline characteristics. Because we include a school-level measure of prevalence, we cluster standard errors at the school level.
>
> […] columns (2) to (4) document that such distance was systematically associated with lower discrepancies between children's and parents' reports. While, in this sub-sample, the average discrepancy between children's and parent's reports was 22.9 p.p., we estimate that it decreased by 1.7 p.p. (significant at the 5% level) with every km away from school, 7.4% of the average discrepancy. [..] **Results support the certifier claim that remoteness is inversely associated with reporting biases, without necessarily being associated with children in employment itself.** […]
>
> All in all, **results lend confidence to the claim that the certifier's sampling strategy might indeed reveal the 'ground truth' when it comes to the local prevalence of child labor.** Moreover, they suggest **children's self-reports in the remote villages surveyed by ENVERITAS are likely representative of those in other villages.** As such, we can **confidently use that data to validate the prevalence of child labor according to different reporting sources in the sample for which the two datasets overlap**, in the next section".

We find no evidence that the more remote the area, the less child labor; on the contrary, we find no relation between our measure of remoteness and children's self-reports (emphasis in **bold**):

> "In Panel A, column (1) documents that distance from school is not associated with children's self-reports. The average end-line prevalence of children in employment within the sub-sample with valid home GPS data was 50.6%; **such prevalence did not systematically increase with the household's distance to the school. This is consistent with the idea that more remote communities do not necessarily feature more children working in cocoa fields.**"

Last, when it comes to differences in the prevalence of domestic work relative to work in cocoa fields, we can only speculate about its potential sources. For our purposes, it is enough to say that domestic work (helping in household chores, caring for siblings) is not classified as child labor, and has not been the target of international campaigns to discourage it – much different to work in cocoa fields. In Section 2.4.1, we highlight (emphasis in **bold**):

> "Importantly, we estimate those relations not only for child labor in cocoa fields, but also, for domestic work (helping with household chores, etc.) – **much less sensitive to social stigma, and which has not been systematically targeted by NGOs and international organizations promoting children and adolescents' rights in the region**. Domestic work provides a counterfactual to assess the claim that discrepancies between parents' and children's reports are indeed driven by social desirability biases, and a placebo test for the claim that village remoteness should decrease the extent of reporting biases by the adults.

520 Galvez Street – CERAS 519
Palo Alto, CA, 94305
United States

> Concretely, we hypothesize that (1) differences in children in employment across parents' and children's reports should be significantly lower for domestic work relative to labor in cocoa fields […]"

3. For the result, it is necessary to add a GPS image when taking the data which shows that the child labor was indeed photographed with this tool which may be shown in a certain color. So, not only the coverage of cocoa area.

We apologize for the confusion the original manuscript might have caused. ENVERITAS does not visualize child labor with aerial imagery; rather, it uses aerial imagery to locate remote communities during harvest season, where and when they can survey adults – minimizing reporting biases either because of lower exposure to previous sensitization campaigns and because they can observe child labor directly. The introduction states (emphasis in **bold**):

> "This paper uses novel data from a cocoa certifier in Côte d'Ivoire to overcome this challenge. ENVERITAS, a global NGO that certifies smallholder coffee and cocoa farms when it comes to agricultural best practices – including the absence of child labor –, draws on satellite imagery to minimize under-reporting. Concretely, **aerial photos allow them (1) to select remote and hard-to-reach communities, where parents typically have not been sensitized by government or NGO campaigns to discourage child labor, averting social desirability biases; and (2) to visit these communities during harvest season, precisely when child labor is very visible, making it easier for enumerators to mark it as present even if parents still fail to report it**. We compare their figures with those obtained from business-as-usual surveys with parents and children in these regions, which partially overlap with the remote villages where ENVERITAS certifies farming practices. Our sample focuses on primary school children, nearly all under 12 years old – for whom any hours in employment are classified as child labor by national legislation."

In Methods and materials, we provide additional details on how ENVERITAS works:

> "The certifier has specialized in reaching small, hard-to-reach and remote farmers, who often cannot even be located by traditional certifiers. To do that, ENVERITAS relies on partnerships to access fresh acquisitions of 50cm-resolution satellite data filtered for quality (with alleged maximum cloud covers of 15%, "crucial for finding (...) farms in cloudy equatorial regions"; Enveritas, 2020), combined with machine learning models to identify specific crops. Additional details on how such models have been successfully used to map coffee-growing households in different geographies can be found in Tran et al. (2021a). In Côte d'Ivoire, the certifier has applied this methodology to identify constraints to quality education and early childhood development – including child labor – in cocoa growing communities (TRECC, 2021).

Stanford | GRADUATE SCHOOL OF EDUCATION

520 Galvez Street – CERAS 519
Palo Alto, CA, 94305
United States

The combination of satellite imagery with machine learning models generates GPS coordinates for each ENVERITAS field team. Pins in Google Maps assign plots to be surveyed to each enumerator. Michael Kra, country lead for ENVERITAS, points out that '*When one surveys farmers, sometimes they do not tell you the truth. The pins will tell us to go where the truth is*'. The idea is that farmers in hard-to-reach communities are less sensitive to social desirability biases. Michael Kra explains: '*We work in very rural communities. The road is often not good, requiring enumerators to use boats to cross rivers, motorcycles, etc. These communities being much less accessible, farmers have been rarely sensitized about child labor. This is why it is easier for them to openly disclose child labor. We can only find these communities because of satellite imagery*'. Appendix A includes a sample satellite picture to illustrate how they are able to locate cocoa plantations based on aerial imagery. The appendix also compiles pictures from enumerators in the field as they survey some of these remote communities, documenting how they often have to cross dirt roads and flooded paths in order to reach them.

ENVERITAS further monitors cocoa harvests in the country with the help of satellite imagery, surveying farmers specifically during harvest season. With harvesting activities ongoing at the time of the survey, the potential presence of child labor becomes apparent: not only it is much harder for parents to falsely deny children in employment when it is more visible, but also, ENVERITAS complements survey data with direct field observation (Tran et al., 2021a). Michael Kra explains how surveying farmers during harvest time make it harder to under-report child labor: '*Enumerators can mark child labor as present even if a parent fails to report it (although this happens in less than 25% of surveys). They can add comments with additional information they have observed in the farm: e.g., 'child is helping, climbing cocoa trees*'."

Thank you once again for your inputs, which have helped us greatly improve the paper.

Stanford | GRADUATE SCHOOL OF EDUCATION

520 Galvez Street – CERAS 519
Palo Alto, CA, 94305
United States

Reviewer #3: This paper studies a very important and pressing question: to what extent do parents and children under-report the incidence of child labor? The study focuses on cocoa-producing communities in Cote d'Ivoire ---- a context where child labor is frequently engaged, partially due to the nature of the cocoa production process (where a small child may still have decent productivity).

This study uses multiple sources of data: parents surveys, children surveys, and novel satellite imagery-based data to study the extent of under-reporting of child labor practices. The study offers convincing evidence that parents systematically under-report the incidence of child labor by a wide margin, whereas children provide somewhat accurate report of child labor.

I have a few comments to improve the paper.

Thank you for the encouraging feedback and for your valuable suggestions, which have helped us greatly improve the paper.

1. The analysis described in section 5.2 and Table 1 seems to use only endline data from the survey. I would have preferred to see this analysis done using the baseline data, as the relation between child-parent reporting discrepancy and distance to school may have been affected by the anti-child labor intervention carried out between baseline and endline? At the very minimum, the authors should provide Table 1 using both baseline and endline data.

Thank you for noting that the endline data indeed might have been affected by the intervention, and apologies for the oversight in the original manuscript. We have now replaced the Table in the main text to focus on the control group of the intervention, removing households targeted by nudges to parents. Our findings are very robust to that sample restriction, despite a smaller number of observations:

Stanford | GRADUATE SCHOOL OF EDUCATION

520 Galvez Street – CERAS 519
Palo Alto, CA, 94305
United States

Table 1: Association between household distance to school, children's self-reported employment, and within-household reporting discrepancies between parents and children

	Children in employment (self-report)	Discrepancy (child - parent)		
	(1)	(2)	(3)	(4)
**Panel A - Work in cocoa fields**				
Distance from school (km)	0.014	-0.014	-0.017**	-0.017***
	(0.014)	(0.012)	(0.007)	(0.006)
Child labor (self-report)		0.633***	1.063***	1.073***
		(0.037)	(0.066)	(0.072)
Average child labor at school			-0.406***	-0.388***
			(0.078)	(0.086)
Child labor × Average child labor			-0.512***	-0.511***
			(0.122)	(0.131)
Sample mean	0.506	0.229	0.229	0.228
R-squared	0.001	0.376	0.471	0.477
Observations	1363	1362	1362	1235
**Panel B - Domestic work**				
Distance from school (km)	-0.003	0.010	0.009	0.000
	(0.007)	(0.012)	(0.011)	(0.009)
Domestic work (self-report)		0.617***	0.523	0.383
		(0.046)	(0.432)	(0.461)
Average domestic work at school			-0.412	-0.485
			(0.474)	(0.524)
Domestic work × Average domestic work			0.136	0.339
			(0.507)	(0.536)
Sample mean	0.886	0.082	0.082	0.088
R-squared	0.000	0.212	0.215	0.243
Observations	1363	1361	1361	1234
Baseline controls	No	No	No	Yes

The reason not to replicate the analysis with baseline data is that the latter was far removed from harvest season – precisely when the prevalence of child labor is much higher (according to ENVERITAS), introducing much stronger incentives for parents to misreport. To that point, in Section 3.1 ('Descriptive statistics based on children's self-reports'), we write:

> "Appendix C documents the aggregate prevalence of children in employment, and that by student characteristics, according to children's self-reports at baseline. 38.1% of children reported to have worked at least one hour in cocoa fields over the previous month. As a point of comparison, by the end of the school year (closer to harvest season), this figure was up to 50% (see Appendix D)."

Moreover, we remain focused on the same endline data that is the subject of the comparisons with the ENVERITAS data in the following section.

520 Galvez Street – CERAS 519
Palo Alto, CA, 94305
United States

2. The analysis described in section 5.2 and Table 1, all regressions assume a linear relation between “distance to school” and “child labor” / “discrepancy. This assumption seems quite unnatural – especially given that Appendix FigureC3 (distribution of distance to school) shows a very long right tale. The majority of students live within 1km of their school. However, a few live very far away: 5km, 15km, or even 20 km.

a. First, the very high distance numbers are hard to believe, as these are primary school children. If the household is indeed 15 or 20 km away, how does the child commute to school daily? Should these observations at the tail end of the distribution be excluded from the regressions in Table 1? I would like to see a robustness Table excluding the very high distance tables.

b. The linear assumption may also be a stretch. Can the authors present alternative specifications using: (1) log distance to school, and (2) dummy variables showing: 0-0.5km, 0.5-1km, 1-2km, beyond 2km?

Thank you for raising this important concern, which is indeed critical to our findings about the relation between household remoteness and reporting discrepancies. We have now introduced two additional analyses that help document the robustness of our findings to outlier observations when it comes to distance to school, and to parametric assumptions of the econometric specification.

In Section 2.4.1 (‘Assessing the certifier claim that remoteness is inversely related to reporting biases’), we write:

> “We assess the sensitivity of our estimates to outlier observations and to specification choices by plotting the relation between reporting discrepancies and distance from the school, dropping observations further than 1.5 km from the school.”

In Section 3.2, we present our findings (emphasis in **bold**):

> “Figure 1 documents that the relation between household remoteness and reporting discrepancies is **not an artifact of outlier observations or specification choices**. Dropping observations further than 1.5 km from the school from the analyses, the binscatter plot shows that discrepancies indeed decline systematically with distance. **If anything, the relation is even stronger within that range**: a linear regression estimates that discrepancies decrease 4.5 p.p. per km, from a baseline of 25% (an effect size significant at the 10% level).”

Stanford | GRADUATE SCHOOL OF EDUCATION

520 Galvez Street – CERAS 519
Palo Alto, CA, 94305
United States

Figure 1: Within-household discrepancies of children in employment in cocoa fields (self- vs. adult reports), by distance from school (in km)

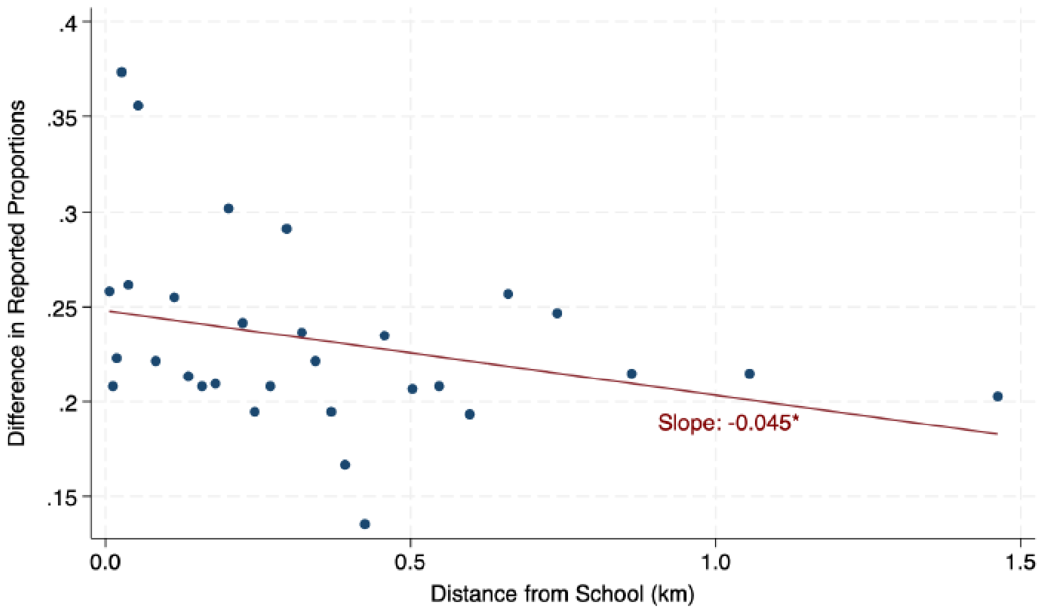

Second, we now also show that the result on the treatment effects of nudges is not driven by outlier observations. In Section 3.4 ('How adult reports can also bias policy evaluations'), we write:

> "Table E1 in Appendix E further documents that results are robust to outlier observations: the coefficient of nudges to parents in column (4) is virtually unchanged if we winsorize distance from school at the 99th percentile."

3. Appendix Figure D2 shows that teachers are just as likely to under-report child labor as parents. An intriguing finding. The main text completely steers away from any discussion of the teachers' reporting. Why?

Thank you for carefully reading through the manuscript and noting that intriguing result. We dedicate less attention to the teacher data because, at the additional follow-up, we were unable to interview children. As such, teachers were not interviewed at the same time as children themselves, introducing comparability challenges. In Figure D2's notes, we mention:

> "Children and parents answer at end line if the child engaged in one hour or more in cocoa plantation activities over the last month, as described in Table B1. **Teachers answer at follow-up** if each of their students worked in cocoa plantation at all over the last school year, as described in Table B1."

If, despite the comparability challenges, the reviewer and the Editor believe it is important to mention the result in the main paper, we would be happy to include it.

520 Galvez Street – CERAS 519
Palo Alto, CA, 94305
United States

4. It’s great to see Section 2.1 with very clear definitions of child labor and how they are supposed to be measured in various frequently cited data. This is great. However, I found the various statistics cited on Page 1 (first three paragraphs of the Introduction) very confusing.

a. “2020 data from the International Labour Organization (ILO) documented that 160 million children worldwide were active workers – 9.6% of all 5-17 year-old children” –-- by definition, among these 160 million, not all count as child labor? Because not all “active workers” between age 5-17 count as child labor. This sentence made it may be there were 160million child labor in 2020 according to ILO estimates. Please clarify.

b. Similarly, for the next two sentences, “in Sub-Saharan Africa, affecting 23.9% of children” –-- does this mean 23.9% of those under 18 engage in child labor (which is very bad)? Or does it mean that 23.9% of those under 18 engage in active work (which may not be bad)?

c. The next sentence: “according to Save the Children, child labor plummeted globally between 2000 and 2016, from 246to 152 million infant workers.” What is “infant workers”? This term is even more confusing. Please also be clear whenever “child” is mentioned in the Introduction, what age range are we referring to, under 18 or under 15?

d. In the 3rd paragraph: “According to NORC… 38% of 5-17 year olds in Côte d’Ivoire reported to have worked in 2018-19(Sadhu et al., 2020); in contrast, the ILO figure for 2016 – based on adult reports for children employment – was only 23%” –-- It seems to me that these discrepancies would be expected if NORCs’ 38% refers to percent of 5-17 year olds working (some of which do not account as child labor, e.g. those aged 15-17 working in non-hazardous tasks), whereas the ILO’s 23% is restricted to child labor.

Generally, the Introduction made it seem like “child labor” means any person under 18 working in any capacity. Please clarify the various statistics cited, which ones are child labor, which ones merely describe under-18 workers.

We agree that the previous version of the manuscript introduced confusion due to the multiplicity of measures mentioned in the introduction. As such, we have now re-written the Introduction to remove ambiguity, as follows:

> “Child labor is a pervasive practice in agriculture, especially in West Africa, where the global cocoa industry sources roughly half of its produce. Defined according to international standards and national legislation, child labor typically characterizes any work by children under the age of 12, and excessive or risky work by 12-17 year-olds (see Section 2.1.1). 2020 data from the International Labor Organization (ILO) documented 160 million child workers worldwide – 9.6% of all 5-17 year-old children (ILO and UNICEF, 2021). Strikingly, that figure was over 2-fold in Sub-Saharan Africa (23.9%). In Côte d’Ivoire, the setting of our study, 15% of the cocoa industry employees that year were children (Sadhu et al., 2020).”

520 Galvez Street – CERAS 519
Palo Alto, CA, 94305
United States

To avoid confusion while still being able to reference commonly cited statistics on child labor levels and trends – albeit measured in different geographies and using different methodologies –, we moved those descriptions to a new subsection within Methods and materials ('How child labor is measured'), as follows:

> "There are striking differences between children's and adults' reports in the few settings where both have been asked independently about children in employment. According to NORC (a research institute based at the University of Chicago), which surveys children directly about the number of hours worked in cocoa fields, 38% of 5-17 year olds in Côte d'Ivoire reported to have worked in 2018-19 (Sadhu et al., 2020); in contrast, the ILO figure for 2016 – based on adult reports for children employment – was only 23% (ILO and UNICEF, 2021). Even worse, different sources tell very different stories about child labor's recent trends. While ILO data indicate a 38% decrease in child labor worldwide since 2000 (Christian Science Monitor, 2019), NORC data record a nearly 65% increase in child labor since 2008-09 (Sadhu et al., 2020).
>
> While these differences are suggestive that official statistics based on adult reports are biased, it is hard to be sure; besides differences in whom is surveyed, discrepancies across surveys might also accrue to differences in their geographical coverage or in the timing of data collection, or to other methodological differences. Moreover, in the absence of verification, it is unclear whether children's self-reports do not suffer from reporting biases too."

Thank you once again for your valuable inputs! We believe the revised manuscript is significantly better as a result of the changes in response to the comments you raised.
