# Supplementary material for: Measuring Child Labor: The Who’s, the Where’s, the When’s, and the Why’s
Source: PLoS One. 2025 Jun 9;20(6):e0322987. doi: 10.1371/journal.pone.0322987 (PMC12148184; doi:10.1371/journal.pone.0322987)
Supplement: Attachment — Submitted filename: Referee Report for PONE-D-24-42933.docx [file pone.0322987.s002.docx]

Referee Report for PONE-D-24-42933

This paper studies a very important and pressing question: to what extent do parents and children under-report the incidence of child labor? The study focuses on cocoa-producing communities in Cote d’Ivoire ---- a context where child labor is frequently engaged, partially due to the nature of the cocoa production process (where a small child may still have decent productivity).

This study uses multiple sources of data: parents surveys, children surveys, and novel satellite imagery-based data to study the extent of under-reporting of child labor practices. The study offers convincing evidence that parents systematically under-report the incidence of child labor by a wide margin, whereas children provide somewhat accurate report of child labor.

I have a few comments to improve the paper.

1. The analysis described in section 5.2 and Table 1 seems to use only endline data from the survey. I would have preferred to see this analysis done using the baseline data, as the relationship between child-parent reporting discrepancy and distance to school may have been affected by the anti-child labor intervention carried out between baseline and endline? At the very minimum, the authors should provide Table 1 using both baseline and endline data.
2. The analysis described in section 5.2 and Table 1, all regressions assume a linear relationship between “distance to school” and “child labor” / “discrepancy. This assumption seems quite unnatural – especially given that Appendix Figure C3 (distribution of distance to school) shows a very long right tale. The majority of students live within 1km of their school. However, a few live very far away: 5km, 15km, or even 20 km.
   a. First, the very high distance numbers are hard to believe, as these are primary school children. If the household is indeed 15 or 20 km away, how does the child commute to school daily? Should these observations at the tail end of the distribution be excluded from the regressions in Table 1? I would like to see a robustness Table excluding the very high distance tables.
   b. The linear assumption may also be a stretch. Can the authors present alternative specifications using: (1) log distance to school, and (2) dummy variables showing: 0-0.5km, 0.5-1km, 1-2km, beyond 2km?
3. Appendix Figure D2 shows that teachers are just as likely to under-report child labor as parents. An intriguing finding. The main text completely steers away from any discussion of the teachers’ reporting. Why?
4. It’s great to see Section 2.1 with very clear definitions of child labor and how they are supposed to be measured in various frequently cited data. This is great. However, I found the various statistics cited on Page 1 (first three paragraphs of the Introduction) very confusing.
   a. “2020 data from the International Labour Organization (ILO) documented that 160 million children worldwide were active workers – 9.6% of all 5-17 year-old children” –-- by definition, among these 160 million, not all count as child labor? Because not all “active workers” between age 5-17 count as child labor. This sentence made it seems like there were 160 million child labor in 2020 according to ILO estimates. Please clarify.
   b. Similarly, for the next two sentences, “in Sub-Saharan Africa, affecting 23.9% of children” –-- does this mean 23.9% of those under 18 engage in child labor (which is very bad)? Or does it mean that 23.9% of those under 18 engage in active work (which may not be bad)?
   c. The next sentence: “according to Save the Children, child labor plummeted globally between 2000 and 2016, from 246 to 152 million infant workers.” What is “infant workers”? This term is even more confusing. Please also be clear whenever “child” is mentioned in the Introduction, what age range are we referring to, under 18 or under 15?
   d. In the 3^rd^ paragraph: “According to NORC… 38% of 5-17 year olds in Côte d’Ivoire reported to have worked in 2018-19 (Sadhu et al., 2020); in contrast, the ILO figure for 2016 – based on adult reports for children employment – was only 23%” –-- It seems to me that these discrepancies would be expected if NORCs’ 38% refers to percent of 5-17 year olds working (some of which do not account as child labor, e.g. those aged 15-17 working in non-hazardous tasks), whereas the ILO’s 23% is restricted to child labor.

Generally, the Introduction made it seem like “child labor” means any person under 18 working in any capacity. Please clarify the various statistics cited, which ones are child labor, which ones merely describe under-18 workers.
